# The hepatocyte IKK:NF-κB axis promotes liver steatosis by stimulating *de novo* lipogenesis and cholesterol synthesis

**DOI:** 10.1016/j.molmet.2021.101349

**Published:** 2021-10-06

**Authors:** Andries Heida, Nanda Gruben, Leen Catrysse, Martijn Koehorst, Mirjam Koster, Niels J. Kloosterhuis, Albert Gerding, Rick Havinga, Vincent W. Bloks, Laura Bongiovanni, Justina C. Wolters, Theo van Dijk, Geert van Loo, Alain de Bruin, Folkert Kuipers, Debby P.Y. Koonen, Bart van de Sluis

**Affiliations:** 1 Departments of Pediatrics, University of Groningen, University Medical Center Groningen, Groningen, the Netherlands; 2 VIB Inflammation Research Center, Ghent University, Ghent, Belgium; 3 Department of Biomedical Molecular Biology, Ghent University, Ghent, Belgium; 4 Department of Biomolecular Health Sciences, Faculty of Veterinary Medicine, Utrecht University, the Netherlands; 5 Faculty of Veterinary Medicine, University of Teramo, Teramo, Italy; 6 Departments of Laboratory Medicine, University of Groningen, University Medical Center Groningen, Groningen, the Netherlands

**Keywords:** Steatosis, Lipid metabolism, Mevalonate pathway, Inflammation, Hypercholesterolemia, Cardiovascular disease

## Abstract

**Objective:**

Obesity-related chronic inflammation plays an important role in the development of Metabolic Associated Fatty Liver Disease (MAFLD). Although the contribution of the pro-inflammatory NF-κB signaling pathway to the progression from simple steatosis to non-alcoholic steatohepatitis (NASH) is well-established, its role as an initiator of hepatic steatosis and the underlying mechanism remains unclear. Here, we investigated the hypothesis that the hepatocytic NF-κB signaling pathway acts as a metabolic regulator, thereby promoting hepatic steatosis development.

**Methods:**

A murine model expressing a constitutively active form of IKKβ in hepatocytes (Hep-IKKβca) was used to activate hepatocyte NF-κB. In addition, IKKβca was also expressed in hepatocyte A20-deficient mice (IKKβca;A20^LKO^). A20 is an NF-κB-target gene that inhibits the activation of the NF-κB signaling pathway upstream of IKKβ. These mouse models were fed a sucrose-rich diet for 8 weeks. Hepatic lipid levels were measured and using [1–^13^C]-acetate *de novo* lipogenesis and cholesterol synthesis rate were determined. Gene expression analyses and immunoblotting were used to study the lipogenesis and cholesterol synthesis pathways.

**Results:**

Hepatocytic NF-κB activation by expressing IKKβca in hepatocytes resulted in hepatic steatosis without inflammation. Ablation of hepatocyte *A20* in Hep-IKKβca mice (IKKβca;A20^LKO^ mice) exacerbated hepatic steatosis, characterized by macrovesicular accumulation of triglycerides and cholesterol, and increased plasma cholesterol levels. Both *De novo* lipogenesis (DNL) and cholesterol synthesis were found elevated in IKKβca;A20^LKO^ mice. Phosphorylation of AMP-activated kinase (AMPK) - a suppressor in lipogenesis and cholesterol synthesis - was decreased in IKKβca;A20^LKO^ mice. This was paralleled by elevated protein levels of hydroxymethylglutaryl-CoA synthase 1 (HMGCS1) and reduced phosphorylation of HMG-CoA reductase (HMGCR) both key enzymes in the cholesterol synthesis pathway. Whereas inflammation was not observed in young IKKβca;A20^LKO^ mice sustained hepatic NF-κB activation resulted in liver inflammation, together with elevated hepatic and plasma cholesterol levels in middle-aged mice.

**Conclusions:**

The hepatocytic IKK:NF-κB axis is a metabolic regulator by controlling DNL and cholesterol synthesis, independent of its central role in inflammation. The IKK:NF-κB axis controls the phosphorylation levels of AMPK and HMGCR and the protein levels of HMGCS1. Chronic IKK-mediated NF-κB activation may contribute to the initiation of hepatic steatosis and cardiovascular disease risk in MAFLD patients.

## Highlights

• The hepatocytic IKK:NF-κB axis is a metabolic regulator.
• Hepatocyte IKK-mediated NF-κB activation drives liver steatosis but not liver inflammation.
• Chronic activation of the hepatocyte NF-κB signaling pathway promotes *de novo* lipogenesis and cholesterol synthesis.
• IKK-mediated NF-κB activation in hepatocytes contributes to MAFLD severity.

## 1 Introduction

Non-alcoholic fatty liver disease (NAFLD), renamed as Metabolic Associated Fatty Liver Disease (MAFLD) [1], is becoming the most common liver disease globally [1]. MAFLD frequently coexists with obesity, type 2 diabetes, and metabolic syndrome and increases the risk of cardiovascular disease [[1], [2], [3]]. Hepatic lipid accumulation in the absence of liver damage and inflammation is the predominant characteristic of benign MAFLD, also referred to as simple steatosis. Simple steatosis can progress to non-alcoholic steatohepatitis (NASH), characterized by steatosis, liver inflammation, and hepatocellular damage. At this stage of MAFLD, the liver is predisposed to develop liver fibrosis, cirrhosis, and hepatocellular carcinoma (HCC) [4]. Compelling evidence indicates a critical role for the activation of the nuclear factor κB (NF-κB) of transcription factors in the progression from simple steatosis to NASH and the other MAFLD-related liver complications, but its contribution to the initiation of hepatic steatosis remains undefined [[5], [6], [7]].

The NF-κB family consists of five members, NF-κB1 (p105/p50), NF-κB2 (100/p52), RelA (p65), Relb and cRel [8]. Activation of the trimeric IκB kinase (IKK) complex is an important step in the canonical NF-κB activation pathway [9]. The IKK complex, composed of NF-κB essential modulator (NEMO) and the subunits IKKα and IKKβ, phosphorylates the inhibitory IκB proteins, including IκBα, leading to proteasomal degradation of these inhibitors [10,11]. This enables the NF-κB subunits to translocate from the cytoplasm to the nucleus, where they activate the transcription of an array of genes involved in many different pathways, including inflammation, cell proliferation, and apoptosis. The gene *A20* (also called TNF alpha-induced protein 3, *TNFAIP3*), encoding a ubiquitin-editing protein, is one of the few NF-κB target genes that act upstream of IKK as a negative feedback loop to terminate NF-κB signaling and protects cells from apoptosis [12]. The NF-κB pathway is chronically activated in NASH and its activation in obese individuals is often associated with higher circulating levels of pro-inflammatory cytokines such as TNF, IL-6, and IL-1β [13,14]. Several studies have indicated that hepatic NF-κB activation is an important player in the progression and initiation of MAFLD. Inhibition of NF-κB activity by hepatocytic silencing of the NF-κB subunit p65 has been shown to protect mice from diet-induced steatosis [15]. Chronic activation of hepatocyte NF-κB in a diet-induced MAFLD model or by overexpressing a constitutively active IKKβ mutant (*Ikk2ca*) in hepatocytes exacerbates hepatic steatosis without concomitant hepatocellular damage or lobular inflammation [16,17]. Furthermore, increased expression of A20 has been identified in steatotic human liver samples [18]. Although these studies suggest a causal relationship between hepatic NF-κB activation and MAFLD development, the contribution of NF-κB activation to the initiation of hepatic steatosis and the underlying mechanism remains unclear.

To study the role of the pro-inflammatory NF-κB signaling pathway in the initiation of hepatic steatosis, we used mice that expressed a constitutively active form of IKKβ exclusively in hepatocytes with or without hepatocytic A20. This study identified a hepatocyte-specific role for the NF-κB signaling pathway in the initiation of MAFLD, distinct from its role as a key regulator of inflammation. We showed that IKK-mediated activation of hepatocyte NF-κB induces *de novo* lipogenesis (DNL) and cholesterol synthesis, leading to hepatic accumulation of triglycerides and cholesterol, accompanied by high plasma cholesterol levels. We further report that the IKK:NF-κB axis controls the phosphorylation levels of 5′AMP-activated protein kinase (AMPK) and hydroxymethylglutaryl-CoA reductase (HMGCR) and the protein levels of hydroxymethylglutaryl-CoA synthase **(**HMGCS1). Thus, the hepatic NF-κB signaling pathway acts as an initiator of hepatic steatosis and cholesterol synthesis, hence may also contribute to cardiovascular disease risk in MAFLD patients.

## 2 Material and methods

### 2.1 Mice

B6(Cg)-Gt(ROSA)26Sor^tm4(*Ikbkb*)/Rsky^/J mice were obtained from Jackson Laboratory, California, USA (#008242) [17,19,20]. To express the constitutively active form of IKKβ specifically in hepatocytes, these mice were crossed with *Alb-Cre*-transgenic mice, which were purchased from Jackson Laboratory (#003574) to obtain mice homozygous for the *Ikk2ca* transgene and hemizygous for the *Alb-Cre* allele (Hep-IKKβca). Previously described mice carrying the floxed *A20* allele [20] were crossed with Hep-IKKβca mice to generate mice expressing IKKβca specifically in hepatocytes in a hepatocyte A20-deficient background (IKKβca;A20^LKO^). All experiments were performed on male mice and all strains were maintained on a C57BL/6J genetic background. Mice were housed individually in climate-controlled rooms with a 12-hour light/12-hour dark cycle and fed *ad libitum* with a standard laboratory show diet (RM1, Special Diet Services, UK) or a carbohydrate-rich diet (33.5% sucrose, 29.9% corn starch, 3.3% Lodex 10 (Research Diets D12450Bi), as indicated. The mice received a carbohydrate-rich diet at an age of 12 weeks. In all experiments, littermates without the *Alb-Cre* allele were used as wild-type controls (WT).

Mice were sacrificed after a 4-hour fast, and tissues were snap-frozen in liquid nitrogen and stored at −80 °C until further analysis. Blood was collected by cardiac puncture, and plasma was collected after centrifugation at 1,000 g for 10 min at 4 °C. Certain cohorts of mice were anesthetized by i.p. injection and subjected to gallbladder cannulation for 30 min as described previously [21]. During bile collection, body temperature was stabilized using an incubator. Bile was stored at −80 °C until further analysis. All animal studies were approved by the Dutch Central Committee for Animal Experiments and the Animal Care and Use Committee of the University of Groningen, The Netherlands. Studies with the liver-specific A20 deficient mice were performed in a pathogen-free animal facility of the VIB Inflammation Research Center. Hepatocyte A20-deficient mice were fed on the carbohydrate-rich diet (33.5% sucrose, 29.9% corn starch, 3.3% Lodex 10 (Research Diets D12450Bi) for 8 weeks, and all experiments were performed according to institutional, national, and European animal regulations.

### 2.2 Glucose tolerance test and insulin levels

A glucose tolerance test (GTT) was performed as previously described [22]. Briefly, mice were fasted overnight and injected intraperitoneal with a low dose of glucose (100 mg/kg). Blood glucose concentrations were measured at indicated time points in a blood drop collected by tail-tip bleeding. At the end of the experiment, an orbital puncture was performed to obtain a blood sample (Crystalchem, #90010/90020) for insulin measurements.

### 2.3 Gene expression analysis

Frozen liver tissue (±100 mg) was homogenized in QIAzol Lysis Reagent (Qiagen), and total RNA was isolated according to the manufacturer's instructions. Two micrograms of total RNA were used for cDNA synthesis, according to the manufacturer (Invitrogen, #28025013) protocols. Twenty nanograms of cDNA were used as a template for quantitative real-time PCR (qRT-PCR) analysis using SYBR Green Master (Roche) and qRT-PCR primers (Supplemental Table 1). QRT-PCR data were analyzed using QuanStudio™ Real-Time PCR software using the delta–delta Ct method, and expression was normalized to *Ppia* expression and expressed as fold change compared to WT mice.

Total RNA used for RNA sequence analysis was isolated from frozen liver tissue using the RNAeasy plus kit (Qiagen, #74134) according to the manufacturer's instructions. Quantity and quality were assessed using nanodrop and gel electrophoreses, respectively. RNA samples were used for library preparation and sequencing, performed and analyzed by Novogene Co. Ltd. Europe. The Genome Reference Consortium Mouse Build 38 Organism (GRCm38) genome and gene model annotation files were downloaded from the genome website browser (NCBI/UCSC/Ensembl) directly. Paired-end clean reads were aligned to the reference genome using HISAT2 software. HTSeq was used to count the read numbers mapped to each gene, including known and novel genes. Reads per kilobase of exon model per million mapped reads of each gene were calculated on the length of the gene and read count mapped to this gene (Supplemental Table 2). Differential expression analysis between two groups (n = 6 per group) was performed using the DESeq2 R package. DESeq2 provides statistical routines for determining differential expression in digital gene expression data using a model based on the negative binomial distribution. The resulting P-values were adjusted using Benjamini and Hochberg's approach for controlling the False Discovery Rate (FDR). Genes with an adjusted P-value <0.05 found by DESeq2 were assigned as differentially expressed.

RNA sequencing data were subsequently used to construct heatmaps and perform GSEA. Heatmaps were constructed based on the z-score calculated from the raw FPKM scores of individual genes per mouse. Gene Set Enrichment Analysis (GSEA) for beta-oxidation was performed using the Gene Ontology ID: GO:0006635 dataset and RNA expression data were ranked according to the log2FC score of gene expression. Enrichment score (ES) and normalized ES (NES) were determined and GSEA was performed using GSEA 4.1.0 software (Broad Institute and UC San Diego).

### 2.4 Immunoblotting

Livers were homogenized in NP-40 buffer (0.1% nonidet P-40 [NP-40], 0.4 M NaCl, 10 mM Tris–HCl [pH 8.0] and 1 mM EDTA, protease and phosphatase inhibitors (Roche)). Bradford-assay (Bio-Rad) was used to determine protein concentrations, and thirty micrograms of protein were used for sodium dodecyl sulphate–polyacrylamide gel electrophoresis (SDS-PAGE) and transferred to Amersham Hybond P PVDF membranes (GE Healthcare: RPN303F). Polyvinylidene difluoride (PVDF) membranes were blocked in 5% milk in tris-buffered saline with 0.01% Tween-20 (TBST) and incubated with antibodies. ChemiDoc XRS + System and Image Lab software version 5.2.1 (Bio-Rad) were used to visualize the proteins. Antibodies used for immunoblotting are shown in Supplemental Table 3.

### 2.5 Histological analysis

For histological analysis, liver samples were fixed in 4% paraformaldehyde or snap-frozen in liquid nitrogen. Paraformaldehyde samples were embedded in paraffin and cut into sections of 4 μm for hematoxylin-eosin (H&E), F4/80, CD11B, B220, CD3, and Pico Sirius-red staining (SR). Frozen samples were cut into sections of 5 μm and used for Oil-Red-O (ORO) staining (Sigma Aldrich, #O0625). Images of the H&E, ORO, and SR sections were obtained with the Leica DM3000 microscope with a mounted Leica DFC420 camera, while F4/80, CD11B, B220, and CD3 staining was imaged using a Hammatusu NanoZoomer (Hamamatsu Photonics, Almere, the Netherlands). Scoring of steatosis and lobular inflammation was done unbiasedly by a board-certified veterinary pathologist of the Dutch Molecular Pathology Center (UU, Utrecht, The Netherlands) according to a method described previously [23]. The immunohistochemically stained liver sections were analyzed as follows: the number of positive cells was counted in 5 (F4/80) or 10 (CD3, B220, CD11b) 40x fields for each case. The average number of positive cells per field was calculated for each case, and an average per group based on genotype was determined finally. Only cells with lymphocyte morphology were counted for CD3, whereas for B220, CD11b, and F4/80 all the positive cells were counted (for F4/80 most of the positive cells were represented by Kupffer cells). Antibodies used for immunohistochemical staining are indicated in Supplemental Table 3.

### 2.6 Cholesterol and triglyceride analysis in plasma and liver

Lipids were extracted from liver homogenates (15% wt/vol in PBS) according to the Bligh & Dyer method [24] and subsequently dissolved in 2% TX100 to determine triglycerides and cholesterol concentration. Total cholesterol (TC) levels were determined using colorimetric assays (Roche, #11489232), and cholesterol standard FS (DiaSys Diagnostic Systems GmbH, #113009910030) was used as a reference. Triglyceride levels were measured using a Trig/GB kit (Roche, #1187771), and Precimat Glycerol standard (Roche, #16658800) was used as a reference.

### 2.7 De novo lipogenesis, chain elongation, fatty acid determination, and cholesterol synthesis

[1–^13^C]-acetate (2% wt/vol) (99 atom %, Isotec/Millipore Sigma) was added to drinking water for five days. D*e novo* lipogenesis (DNL) and fatty acyl chain elongation were determined as described previously [25,26]. Blood spots were collected every 12 h via tail bleeding on filter paper to determine the fractional cholesterol synthesis rates. TC was extracted from blood spots using ethanol/acetone (1:1 v/v). Cholesterol synthesis measurements were performed as previously described [26].

### 2.8 Fatty acid oxidation

Acylcarnitines concentrations were measured in liver homogenate samples, as previously described [27].

### 2.9 Fast-performance liquid chromatography (FPLC) and targeted proteomics

Individual plasma samples were fractionated by FPLC, as previously described [28]. The samples were pooled and the following fractions 36–40, 41–45, 46–50, and 50–55 were used for targeted liquid chromatography-mass spectrometry analysis to determine Apolipoprotein B (ApoB) protein in FPLC fractions, as previously described [28].

### 2.10 Liver enzymes, ATP concentration, and plasma bile acids

Alanine aminotransferase (ALT) and aspartate aminotransferase (AST) were determined in plasma using a clinical chemistry analyzer (Cobas 6000, Roche Diagnostics) with standard reagents (Roche Diagnostics). Plasma bile acid concentrations were determined according to the protocol provided by the manufacturer (Crystal Chem, #80471). ATP concentrations were determined in liver homogenates according to the manufacturer's protocol (Roche, #11699695001) and were normalized to total protein concentration (Biorad, #5000113 and #5000114).

### 2.11 Statistics

All data were presented as the mean ± SEM. Statistical analyses were performed using GraphPad version 8.4.0 Software, and an unpaired 2-tailed Student's *t*-test was used to compare differences between any two groups. *P*-value of less than 0.05 was considered statistically significant: ∗*P* < 0.05, ∗∗*P* < 0.01, and ∗∗∗*P* < 0.001.

## 3 Results

### 3.1 Chronic activation of the hepatocytic NF-κB signaling pathway leads to steatosis

**Figure 1:**
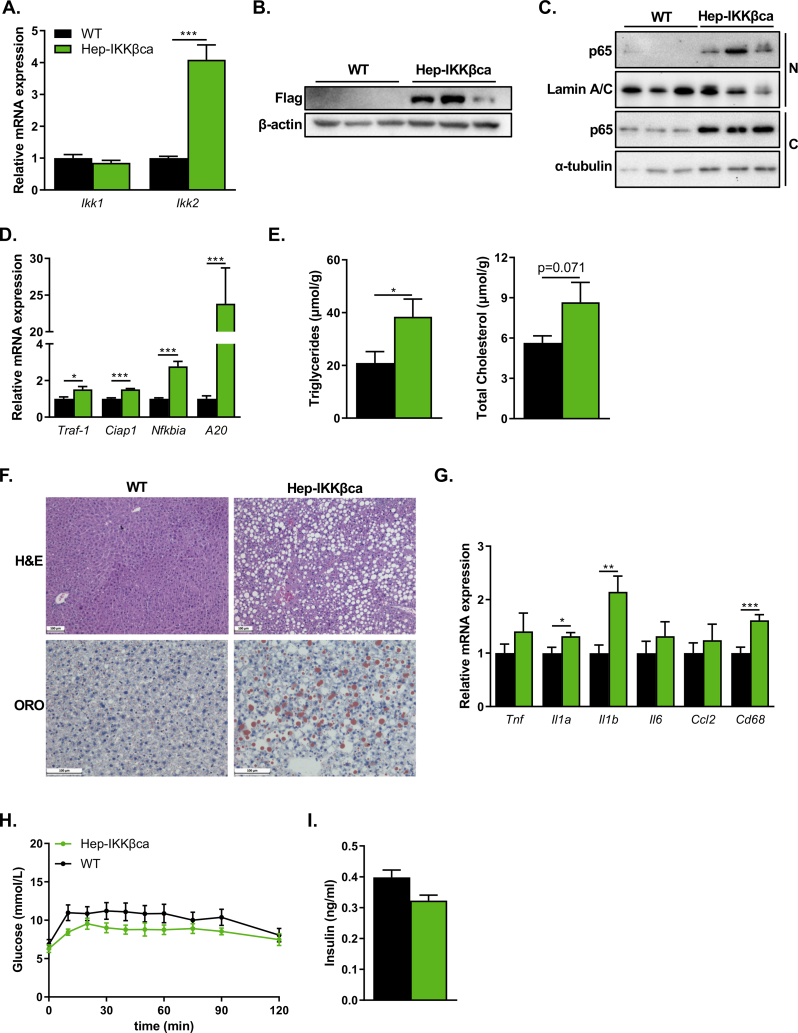
**Hepatocytic IKK-mediated NF-κB activation causes liver steatosis but not inflammation in the carbohydrate-rich diet-fed mice. (A)** Relative liver mRNA expression of *Ikk1* and *Ikk2* in livers of WT and Hep-IKKβca mice fed a standard chow diet as determined by qRT-PCR (n = 15–16). The primers used to analyze *Ikk2* mRNA levels recognized mRNA of endogenous *Ikk2* gene and *Ikk2ca* transgene. **(B)** Protein abundance of IKKβca was determined by immunoblotting using an anti-FLAG antibody. **(C)** Cytosolic (C) and nuclear (N) levels of the NF-κB subunit p65 as determined by immunoblotting. Lamin A/C and α-tubulin were used as markers for nuclear and cytosolic fraction, respectively **(D)** Relative mRNA expression of NF-κB targets genes (n = 6). **(E)** Hepatic triglyceride and cholesterol concentrations (n = 15–16). **(F)** Hematoxylin and eosin (H&E) and Oil-Red-O (ORO) staining of livers of WT and Hep-IKKβca mice fed the carbohydrate-rich diet. Representative image per group is shown. Scale bars represent 100 μm. **(G)** Relative liver mRNA expression of inflammatory markers (n = 15–16). **(H)** Glucose tolerance test (GTT) and **(I)** plasma insulin levels at t = 120 of the GTT in WT and Hep-IKKβca mice (n = 7). Data are presented as mean ± SEM, ∗P < 0.05, ∗∗P < 0.01, ∗∗∗P < 0.001 as determined by Student's *t*-test.

To evaluate the contribution of the pro-inflammatory NF-κB signaling pathway in the development of MAFLD, mice expressing a constitutively active IKKβ mutant in hepatocytes (Hep-IKKβca) were generated. Transgenic mice carrying cDNA encoding Flag-tagged IKKβ containing two serine to glutamate substitutes (S177E, 181E) in the kinase domain of IKKβ [19] were crossed with *Alb*-*cre* transgenic mice to remove the *loxP*-flanked STOP cassette in order to express the *Ikk2ca* transgene in hepatocytes. The hepatic expression of IKKβ was confirmed using quantitative RT-PCR (qRT-PCR) (Figure 1A) and immunoblotting using an antibody directed against the Flag-tag (Figure 1B). IKK-mediated NF-κB activation results in translocation of the subunit p65 from the cytoplasm to the nucleus to transactivate the expression of NF-κB-target genes [8]. Therefore, we analyzed the nuclear levels of hepatic p65 in wildtype (WT) and Hep-IKKβca mice. Nuclear p65 levels were increased in Hep-IKKβca mice compared to WT mice (Figure 1C). In addition, cytosolic p65 levels were increased in Hep-IKKβca compared to WT mice (Figure 1C), which conforms to previous observations [19]. In line with the increased nuclear p65 levels, the expression of the NF-κB targets genes: *Traf-1*, *Ciap1*, *Nfkbia (Ikba),* and *A20* showed significant upregulation in Hep-IKKβca mice (Figure 1D). Body and liver weight and hepatic lipid levels were not different between Hep-IKKβca and WT mice fed a standard chow diet (Figure S1A–D). Histological examination of the livers did not identify liver steatosis, inflammation, and damage in Hep-IKKβca and WT mice (Figure S1E). Altogether, these data indicate successful activation of the NF-κB signaling pathway in hepatocytes, and chronic activation of this pathway does not result in an overt liver phenotype.

In contrast to our observations (Figure S1D and E), hepatic IKKβca expression has previously been shown to result in increased hepatic triglyceride (TG) levels [17]. These phenotypic differences prompted us to stimulate hepatic *de novo* lipogenesis (DNL) by feeding the mice a carbohydrate-rich diet (33.5% sucrose, 29.9% corn starch, 3.3% Lodex 10) [29] for 8 weeks. Feeding the mice the carbohydrate-rich diet resulted in a significant increase in hepatic TG concentration in Hep-IKKβca mice compared to WT mice (Figure 1E), which was confirmed by ORO staining (Figure 1F). In addition, the hepatic cholesterol concentrations tended to be elevated in Hep-IKKβca mice (Figure 1E). Although IKK-mediated NF-κB activation is generally strongly associated with inflammation, the hepatic mRNA levels of the cytokine *Il1b* and the macrophage marker *CD68* were only mildly elevated in Hep-IKKβca mice, while no differences in the expression of other inflammatory markers, such as *Tnf*, *Ila*, *Il6*, *Ccl2* were observed (Figure 1G). Histological examination of the livers did not reveal a substantial difference in the number of inflammatory foci between WT and Hep-IKKβca mice (Figure 1F, Table S4). Furthermore, immunohistochemical staining for the macrophage marker F4/80, monocyte-derived macrophage (MdM) marker CD11b, B-cell marker B220, and T-cell marker CD3 (Figure S2A and B) revealed no difference in liver inflammation. The increased hepatic activity of IKK can lead to glucose intolerance and insulin resistance [30], but Hep-IKKβca mice did not display any glucose intolerance, as determined by GTT (Figure 1H). Plasma insulin levels at the end of the GTT showed no difference between the groups (Figure 1I). Furthermore, no differences in the expression of gluconeogenic genes between Hep-IKKβca and control mice (Figure S2C) were observed. Overall, these data suggest that chronic activation of the NF-κB signaling pathway by hepatocytic expression of IKKβca sensitizes mice to develop hepatic steatosis without causing severe inflammation and glucose intolerance.

### 3.2 Steatosis in Hep-IKKβca mice is not caused by altered expression of genes related to lipogenesis, cholesterol synthesis, or fatty acid oxidation

**Figure 2:**
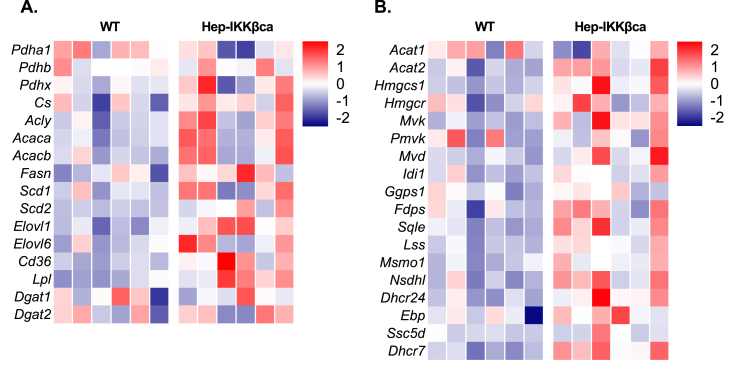
**Hepatocytic NF-κB activation by IKKβca does not induce the expression of lipogenic and cholesterogenic genes. (A)** Heatmap presenting z-score normalized mRNA expression (determined by RNA-seq analysis) of hepatic fatty acid synthesis and **(B)** cholesterol synthesis genes in WT and Hep-IKKβca mice fed the carbohydrate-rich diet (n = 6).

Next, using RNA-seq analysis, we compared the transcriptomes of Hep-IKKβca livers with WT livers to elucidate the mechanism underlying the steatotic phenotype of Hep-IKKβca mice fed the carbohydrate-rich diet. In contrast to a previous study [17], we found no significant differences in the expression of lipogenic genes between WT and Hep-IKKβca mice (Figure 2A). In addition, cholesterogenic genes were not differentially expressed upon hepatocytic IKKβca expression (Figure 2B). Pharmacological inhibition of IKKβ has been shown to prevent hepatic steatosis and inflammation by improving β-oxidation [31]. To assess whether β-oxidation is affected in Hep-IKKβca mice, we analyzed the expression of genes related to fatty acid oxidation. No marked expression differences were observed between WT and Hep-IKKβca mice (Figure S3A and B). In line with this observation, the content of acylcarnitines in livers of Hep-IKKβca mice was not affected (Figure S3C). These findings indicate that hepatic lipid accumulation in Hep-IKKβca mice is not caused by impaired β-oxidation or altered expression of lipogenic and cholesterogenic genes.

### 3.3 Hepatic A20 deficiency exacerbates steatosis in Hep-IKKβca mice

**Figure 3:**
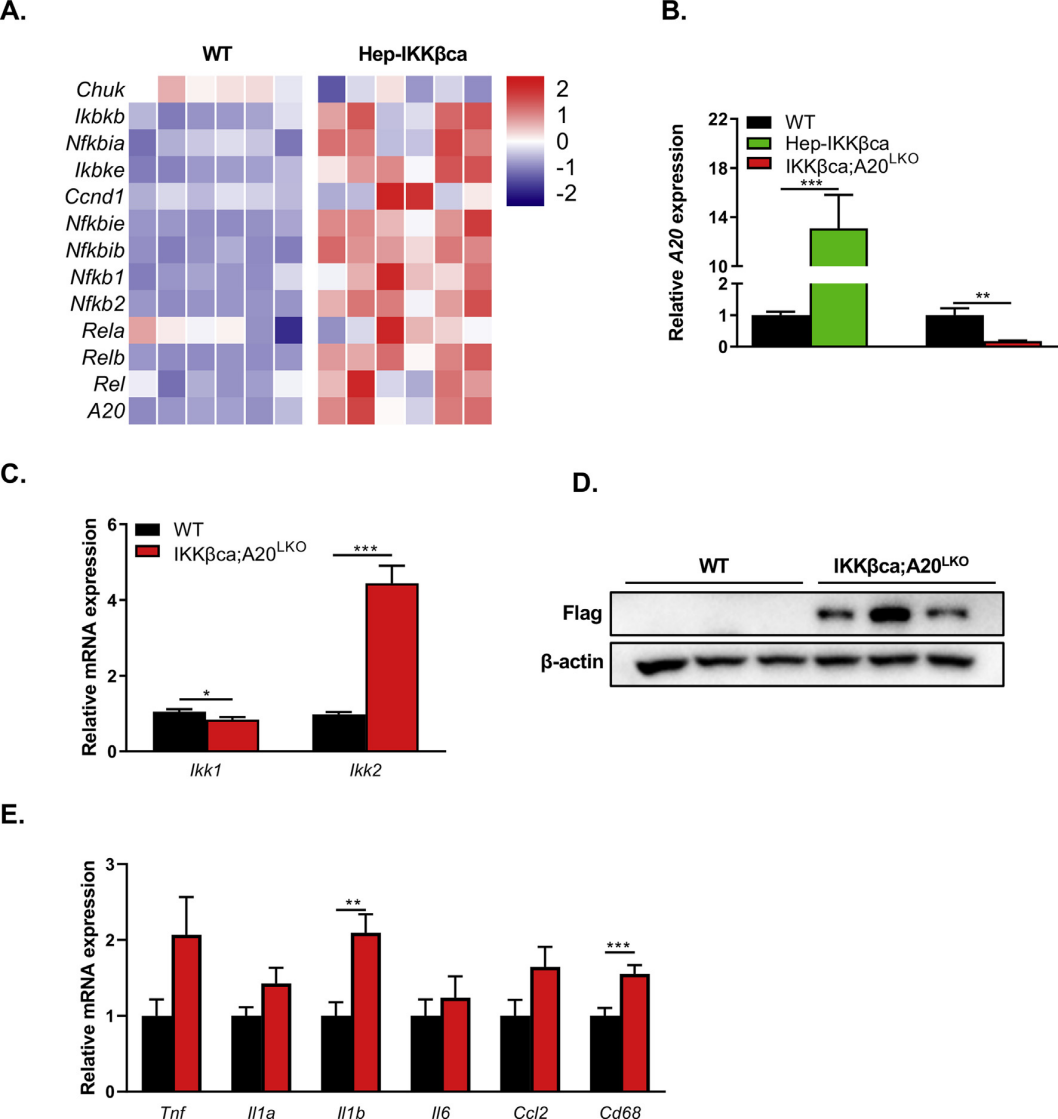
**Hepatocyte-A20 does not restrain liver inflammation in Hep-IKKβca mice.(A).** Heatmap presenting z-score normalized mRNA expression (determined by RNA-seq analysis) of NF-κB target genes in the livers of WT and Hep-IKKβca mice fed the carbohydrate-rich diet (n = 6). **(B)** Relative hepatic *A2*0 mRNA expression in Hep-IKKβca and IKKβca;A20^LKO^ mice compared to WT mice (n = 5–6). **(C)** Relative *Ikk1* and *Ikk2* mRNA expression in livers of WT and IKKβca;A20^LKO^ mice (n = 15–16). **(D)** Hepatic protein abundance of IKKβca using anti-FLAG antibody. **(E)** Hepatic mRNA expression of pro-inflammatory markers in WT and IKKβca;A20^LKO^ mice were determined by qRT-PCR analysis (n = 15–16). Data are presented as mean ± SEM, ∗P < 0.05, ∗∗P < 0.01, ∗∗∗P < 0.001 as determined by Student's *t*-test.

**Figure 4:**
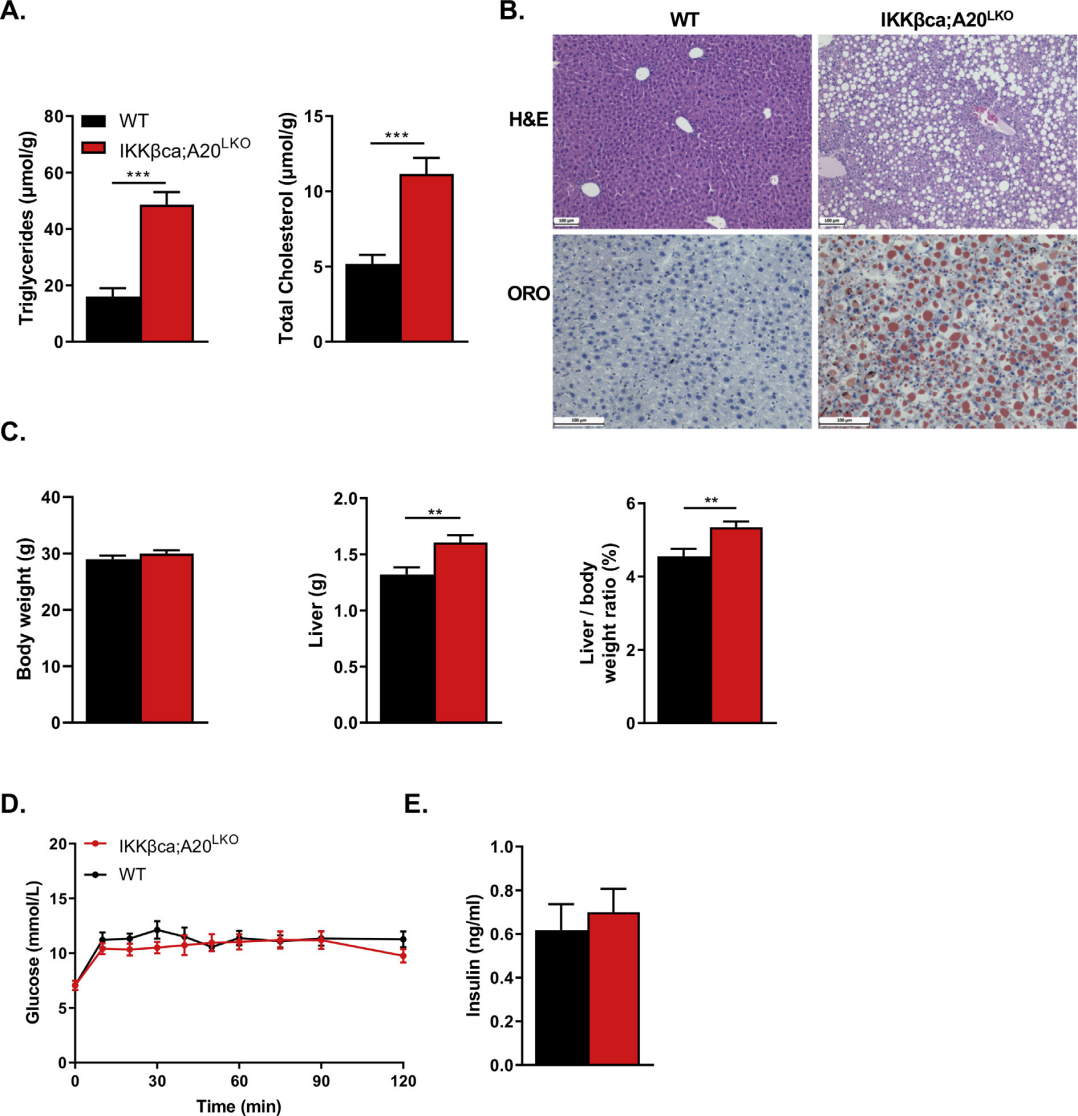
**Hepatocytic A20 deficiency exacerbates steatosis in Hep-IKKβca mice. (A)** Hepatic triglyceride and cholesterol concentrations in WT and IKKβca;A20^LKO^ mice fed the carbohydrate-rich diet (n = 15–16). **(B)** Representative picture of H&E and ORO stainings of livers of WT and IKKβca;A20^LKO^ mice, scale bars represent 100 μm. **(C)** Bodyweight, liver weight, and liver to body weight ratios of WT and IKKβca;A20^LKO^ (n = 15–16). **(D)** Glucose tolerance test (GTT) and **(E)** plasma insulin levels at t = 120 of the GTT in WT and IKKβca;A20^LKO^ mice (n = 7). Data are presented as mean ± SEM, ∗∗P < 0.01, ∗∗∗P < 0.001 as determined by Student's *t*-test.

In agreement with the study of Lu et al. [17], hepatic IKKβca expression induced the expression of numerous genes of the NF-κB pathway, including the NF-κB subunits *Relb* and *NF-κB1* (Figure 3A). Moreover, the NF-κB target gene *A20,* which acts upstream of IKK to terminate NF-κB activation [32], was significantly increased (Figure 3A). This increase was confirmed by qRT-PCR analysis; we found that the *A2*0 mRNA levels were 13-fold higher (Figure 3B). An increase in *A2*0 mRNA levels was also seen in Hep-IKKβca mice fed a standard chow diet (Figure 1D), suggesting that the NF-κB-mediated *A20* transactivation is diet-independent. We hypothesized that this marked increase in *A2*0 mRNA levels dampens liver inflammation in Hep-IKKβca mice. To examine the role of A20 in preventing liver inflammation in Hep-IKKβca mice, we ablated *A20* in hepatocytes of Hep-IKKβca mice (IKKβca;A20^LKO^ mice) by crossing the Hep-IKKβca mice with *A20* floxed mice [20]. Although we successfully deleted hepatic *A20* in Hep-IKKβca mice (Figure 3B–D), IKKβca;A20^LKO^ mice fed the carbohydrate-rich diet showed no marked liver inflammation compared to WT mice, as determined by qRT-PCR analysis (Figure 3E) and histological and immunohistochemical characterization of liver tissues (Table S5, Figure S4A and B). However, the number of CD11b cells (MdM marker) was significantly increased in liver sections of IKKβca;A20^LKO^ mice (Figure S4A and B). A study showed a positive correlation of the number of CD11b cells in livers with MAFLD progression [33]. In line with these results, levels of livers TG and cholesterol concentrations were significantly elevated in IKKβca;A20^LKO^ mice compared to WT (Figure 4A). This hepatic lipid accumulation (Figure 4A and B) was accompanied by an increase in liver weight in IKKβca;A20^LKO^ mice, whereas body weight did not differ between the mice (Figure 4C). The TG and cholesterol concentrations in IKKβca;A20^LKO^ livers were 2.6-fold and 2.1-fold higher (Figure 4A) versus 1.8-fold and 1.5-fold higher in Hep-IKKβca livers compared to WT littermates, respectively (Figure 1E). Hepatic A20-deficient mice (A20^LKO^) without transgenic expression of IKKβca did not display liver steatosis when fed the carbohydrate-rich diet, as no difference in liver TG concentrations were evident (Figure S4C). Hepatic loss of A20 did not increase NF-κB activity, illustrated by the similar induction of the expression of NF-κB genes, including the NF-κB target genes *Nfkbia* (*Ikba*), *Nfkbie* (*Ikbe*), *Nfkbib* (*Ikbb*) (Figure S4D). In addition, no differences in the expression of gluconeogenic genes (Figure S5A), and glucose and insulin response were seen between IKKβca; A20^LKO^ and control mice (Figure 4D and E).

Taken together, these data strongly suggest that constitutive activation of the IKK complex in hepatocytes increases the susceptibility to develop steatosis, which is aggravated by hepatocyte A20 deficiency, likely due to a further increase in IKK complex activity but not by a further increase in NF-κB activity.

### 3.4 The IKK:NF-κB axis controls lipogenesis and cholesterol synthesis at the post-translational level

**Figure 5:**
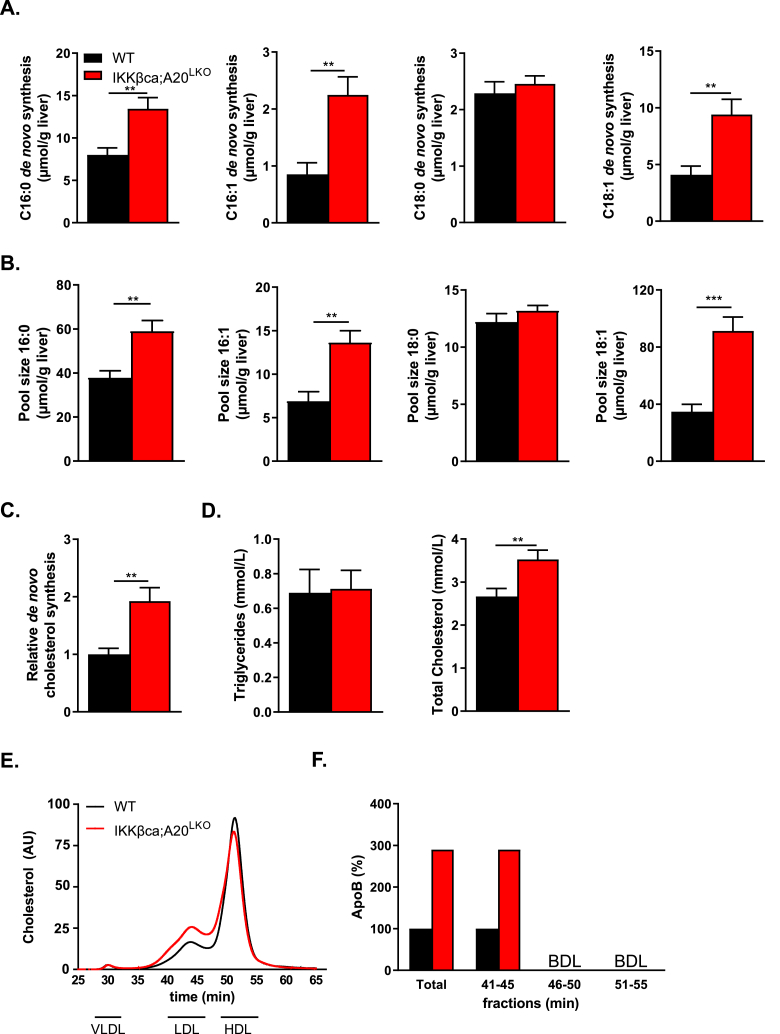
**Steatosis in IKKβca;A20**^**LKO**^**mice is associated with increased *de novo* lipogenesis and cholesterol synthesis. (A)** Absolute *de novo* synthesis of palmitate (C16:0), palmitoleic acid (C16:1), stearate (C18:0), and oleate (C18:1) from livers of WT and IKKβca;A20^LKO^ mice (n = 10). **(B)** Pool size of 16:0, C16:1, C18:0, and C18:1. **(C)** Relative hepatic cholesterol synthesis in WT and IKKβca;A20^LKO^ mice (n = 10). **(D)** Plasma total cholesterol and triglyceride concentrations (n = 15–16). **(E)** Total cholesterol levels of FPLC fractioned plasma pools of WT and IKKβca;A20^LKO^ mice (n = 4–5). Total cholesterol levels were expressed in arbitrary units (AU). **(F)** ApoB levels in pooled FPLC fractionated plasma depicted in (E) was determined by targeted proteomics, indicated as a percentage relative to the total ApoB levels in these fractions. BDL = below detection limit. Data are presented as mean ± SEM, ∗∗P < 0.01, ∗∗∗P < 0.001 as determined by Student's *t*-test.

To understand mechanistically the hepatic steatosis development upon chronic IKK-mediated NF-κB activation, we quantified hepatic DNL and cholesterol synthesis by allowing incorporation of [1–^13^C]-acetate into fatty acids and cholesterol for five days, followed by mass isotopomer distribution analysis (MIDA) [34], in IKKβca;A20^LKO^ mice and WT mice. *De novo* synthesis of palmitoleic (C16:1) and oleic acid (C18:1), the primary fatty acids incorporated in TG, cholesterol esters, and phospholipids were increased in IKKβca;A20^LKO^ mice compared to WT mice (Figure 5A). The pools of palmitic acid (C16:0), palmitoleic (C16:1), and oleic acid (C18:1) were elevated in IKKβca;A20^LKO^ mice (Figure 5B). Furthermore, cholesterol synthesis in IKKβca;A20^LKO^ mice was 2-fold higher compared to WT mice (Figure 5C). This increase in cholesterol synthesis was not only reflected by higher hepatic cholesterol levels (Figure 4A) but was also associated with elevated total plasma cholesterol levels (Figure 5D).

The latter was mainly due to an increase in low-density lipoprotein (LDL) cholesterol levels, as determined by FPLC analysis (Figure 5E) and targeted proteomics analysis of ApoB protein content in the FPLC fractions (Figure 5F). No difference in plasma TG was seen between WT and IKKβca;A20^LKO^ mice (Figure 5D). Despite the marked increase in lipogenesis and cholesterol synthesis in IKKβca; A20^LKO^ mice, the expression of genes related to these pathways (e.g., *Acly, Acaca, Hmgcs1, Hmgcr, Sqle*) were not different between WT and IKKβca;A20^LKO^ mice (Figure S5B and C). Furthermore, the elevation in plasma cholesterol levels in IKKβca;A20^LKO^ mice could not be explained by a strong protein reduction in any of the well-known lipoprotein receptors, LDLR, LRP1, and SR-BI (Figure S6A and B).

The *de novo* synthesis of palmitoleic (C16:1) also showed a significant increase in Hep-IKKβca mice, whereas the *de novo* synthesis of oleic acid (C18:1) and cholesterol synthesis tended to be elevated (Figure S7A and B). This modest increase in DNL and cholesterol synthesis is in line with the less severe steatotic phenotype of Hep-IKKβca mice compared to IKKβca;A20^LKO^ mice.

**Figure 6:**
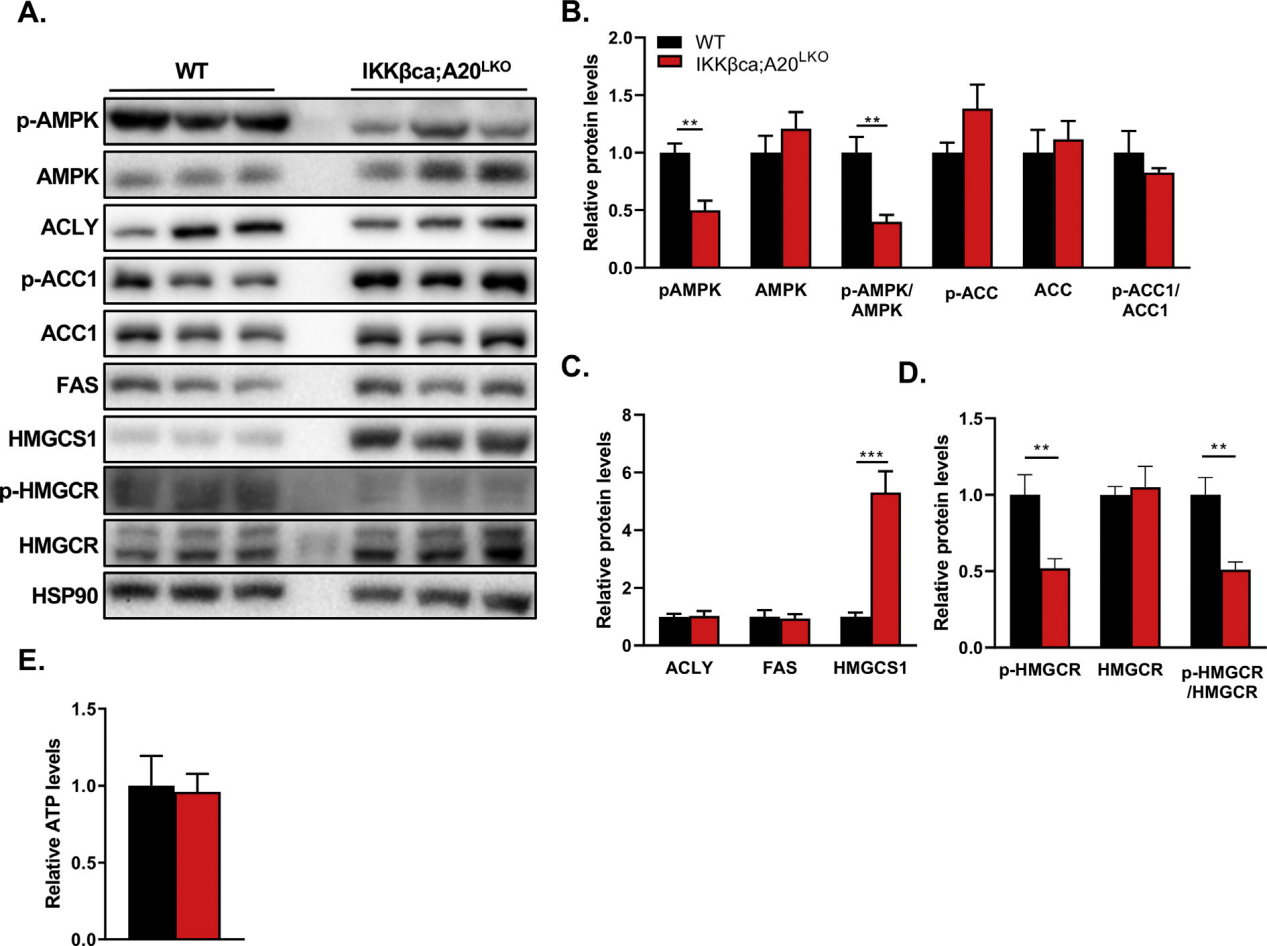
**Hepatocytic NF-κB activation reduces the phosphorylation levels of hepatic AMPK and HMGCR, and increases the protein levels of HMGCS1 (A)** Representative immunoblot of the hepatic levels of the indicated proteins in WT and IKKβca;A20^LKO^ mice (p-AMPK = phosphorylated AMPK, p-ACC = phosphorylated ACC, p-HMGCR = phosphorylated HMGCR)**. (B)** Quantification of hepatic AMPK, p-AMPK, and p-AMPK/AMPK levels, p-ACC, ACC1, and p-ACC/ACC1 levels in WT and IKKβca;A20^LKO^ mice as determined by immunoblotting (n = 5). **(C)** and **(D)** Quantification of the levels of ACLY, FAS, HMGCS1, p-HMGCR, HMGCR, and p-HMGCR/HMGCR in IKKβca;A20^LKO^ and WT mice as determined by immunoblotting (n = 5). **(E)** Relative ATP levels in livers of IKKβca;A20^LKO^ compared to WT mice. Data are presented as mean ± SEM, ∗∗P < 0.01, ∗∗∗P < 0.001 as determined by Student's *t*-test.

AMPK acts as a central player in controlling hepatic lipogenesis and cholesterol synthesis [35,36], and phosphorylation of AMPK inhibits lipogenesis and cholesterol synthesis [[37], [38], [39]]. In line with elevated DNL (Figure 5A), we found a 2-fold decrease in the phosphorylation levels of AMPK in livers of IKKβca;A20^LKO^ mice compared to WT mice (Figure 6A and B). However, this did not affect the phosphorylation status of acetyl-CoA carboxylase 1 (ACC1) in IKKβca;A20^LKO^ mice or the protein levels of fatty acid synthase (FAS), both well-known downstream targets of AMPK (Figure 6A−C). Moreover, ATP citrate lyase (ACLY) protein levels − a key player in lipogenesis − were not affected upon IKK-mediated NF-κB activation in IKKβca;A20^LKO^ mice (Figure 6A and C), indicating that IKK did not control lipogenesis by promoting the protein stability of ACLY, as shown previously [40]. However, the protein expression of 3-hydroxy-3-methylglutaryl-CoA synthase 1 (HMGCS1) was increased ±5-fold in livers of IKKβca;A20^LKO^ mice (Figure 6A and C), and the phosphorylation status of 3-hydroxy-3-methylglutaryl-CoA reductase (HMGCR) at Serine 871 was significantly decreased in livers of IKKβca;A20^LKO^ mice compared to WT mice (Figure 6A and D). The reduced levels of phosphorylated AMPK and HMGCR in IKKβca;A20^LKO^ livers could not be explained by reduced ATP levels (Figure 6E). Phosphorylated AMPK has been shown to phosphorylate HMGCR, thus inhibiting its activity [35,39]. This suggests that the increased HMGCR activity and the elevated HMGCS1 levels observed in the current study may explain the increased cholesterol synthesis in IKKβca;A20^LKO^ mice.

### 3.5 Chronic hepatocytic activation of the IKK:NF-κB axis leads to liver inflammation but does not cause other MAFLD-associated liver complications in middle-aged mice

**Figure 7:**
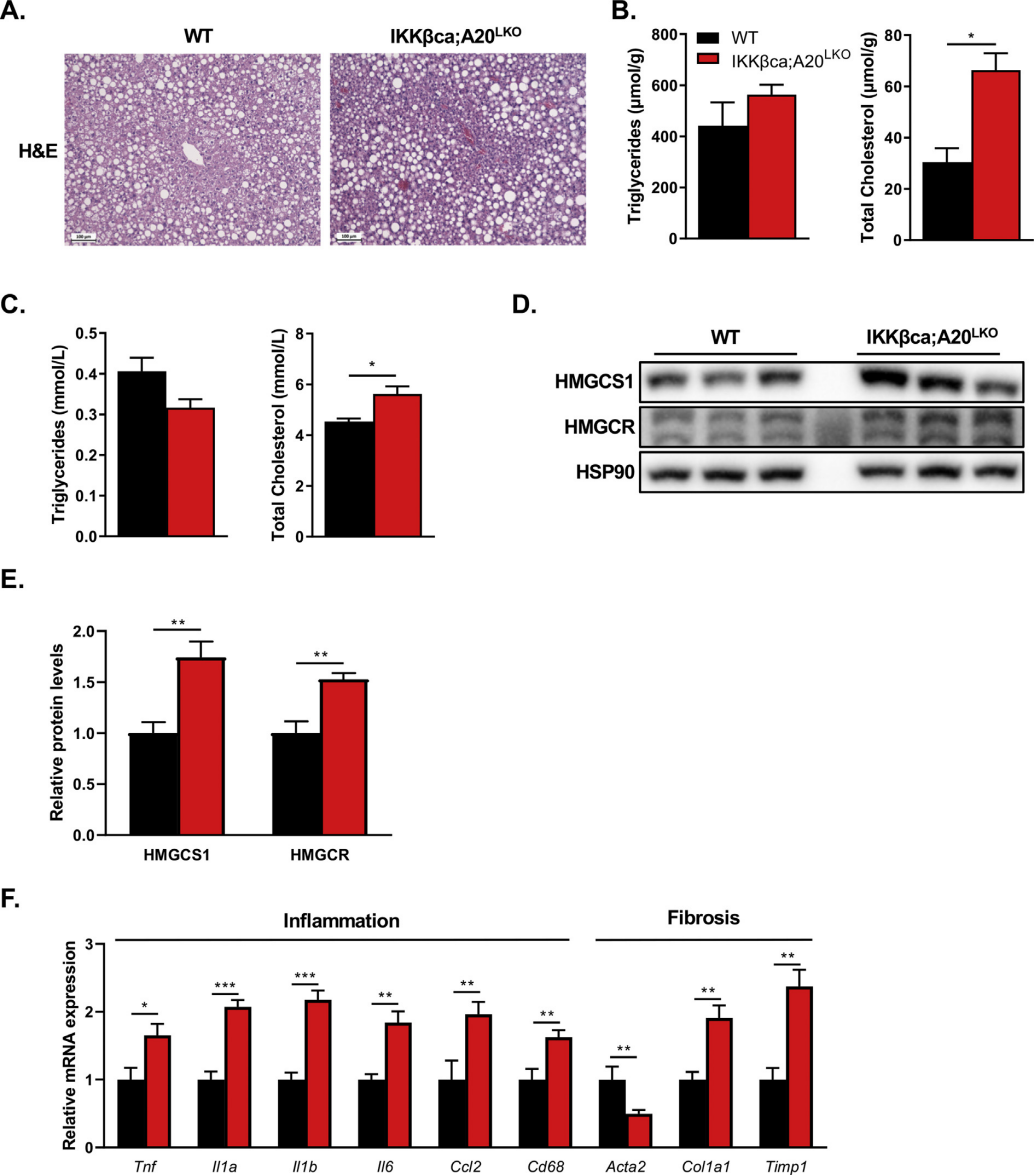
**Hepatocytic activation of the NF-κB signaling pathway drives steatosis and liver inflammation in middle-aged mice. (A)** Representative picture of H&E staining of livers of WT and IKKβca;A20^LKO^ mice fed the carbohydrate-rich diet, scale bars represent 100 μm. **(B)** Hepatic and **(C)** plasma cholesterol and triglyceride concentrations. **(D)** Representative Immunoblot and (**E**) quantification of the hepatic levels of the indicated proteins in IKKβca;A20^LKO^ and WT mice (n = 5). **(F)** Relative hepatic mRNA levels of pro-inflammatory and fibrotic markers in WT and IKKβca;A20^LKO^ mice as determined by qRT-PCR analysis (n = 7–12). Data are presented as mean ± SEM, ∗P < 0.05, ∗∗P < 0.01, ∗∗∗P < 0.001 as determined by Student's *t*-test.

Steatosis can progress to severe liver complications, such as hepatitis, fibrosis, cirrhosis, and HCC. Compelling evidence exists for a significant role of the NF-κB signaling pathway in all these pathologies [17,41]. To investigate the contribution of chronic hepatic IKK-mediated NF-κB activation to MAFLD progression, we aged WT and IKKβca;A20^LKO^ mice to one year of age. Mice started receiving the carbohydrate-rich diet at the age of 12 weeks. We found no differences in body and liver weight, fasted (6 h) blood glucose levels (Figure S8A–D), and both groups developed macrovesicular steatosis (Figure 7A, Table S6). Although liver TG levels were not different between the groups, hepatic cholesterol concentrations were significantly increased in IKKβca;A20^LKO^ mice compared to WT mice (Figure 7B). Similar to young mice, plasma cholesterol levels were elevated in 1-year-old IKKβca;A20^LKO^ mice, but no differences in plasma TG levels were seen (Figure 7C). This increase in hepatic and plasma cholesterol levels was paralleled with a significant increase in HMGCS1 and HMGCR protein levels in IKKβca;A20^LKO^ compared to WT mice (Figure 7D and E). The mRNA expression of the ATP-binding cassette (ABC) transporters G5 (*Abcg5*) and G8 (*Abcg8*), which form a heterodimer to facilitate biliary cholesterol secretion, were not affected in IKKβca;A20^LKO^ mice (Figure S8E), suggesting that the elevated hepatic cholesterol levels in IKKβca;A20^LKO^ mice are not due to impaired biliary cholesterol secretion. The plasma bile acid concentrations between the two groups showed no significant difference (Figure S8F), which indicates that bile acid metabolism is not affected upon chronic IKK-mediated NF-κB activation. The expression of pro-inflammatory cytokines, such as *Tnf*, *Il1a*, *Il1b*, *Il6* (Figure 7F) in livers of middle-aged IKKβca;A20^LKO^ mice were increased compared to control livers. The mRNA levels of the fibrotic markers, *Col1a1* and *Timp1,* were also elevated (Figure 7F), but no significant signs of liver fibrosis were observed by Sirius-red staining of liver sections of WT and IKKβca;A20^LKO^ mice (Figure S8G and H). The plasma levels of the liver enzymes ALT and AST (Figure S8I and J) were not different between the two groups, indicating that chronic IKK-mediated NF-κB activation does not lead to impaired liver function or liver damage. Histological examination did not identify any other severe liver complication, such as liver cirrhosis or cancer. Overall, these results indicate that hepatic activation of the NF-κB signaling pathway not only promotes the progression of liver steatosis, likely by increased cholesterol synthesis but also drives liver inflammation at a later stage of MAFLD.

## 4 Discussion

Hepatic activation of the NF-κB signaling pathway has been observed in many stages of MAFLD [42], but the role of this pathway in the initiation of liver steatosis remains ill-defined. In this study, we show that increased activity of IKK, an upstream activator of the pro-inflammatory NF-κB signaling pathway, sensitizes mice to develop simple steatosis. This phenotype was exacerbated by the hepatocytic loss of A20, an upstream inhibitor of the IKK complex, as IKKβca;A20^LKO^ mice exhibit hepatic accumulation of both TG and cholesterol. In addition, plasma levels of atherogenic LDL cholesterol were increased upon hepatocytic *A20* ablation in Hep-IKKβca mice. We demonstrate that the IKK:NF-κB axis controls DNL and cholesterol synthesis post-translationally by regulating the phosphorylation levels of AMPK and HMGCR and the protein levels of HMGCS1. Furthermore, in middle-aged mice, prolonged activation of the NF-κB signaling pathway also drives the severity of MAFLD, illustrated by low-grade liver inflammation. Our data demonstrate a hepatocyte-specific role for the IKK:NF-κB axis in MAFLD distinct from its role as a key regulator of inflammation. Thus, we suggest that the NF-κB signaling pathway is a key player not only in the progression but also in the initiation of MAFLD.

A hallmark of obesity is low-grade systemic inflammation, caused primarily by the secretion of inflammatory cytokines by enlarged adipose tissues [5,43]. Besides adipose tissues, microbiota-derived lipopolysaccharide (LPS) also participates in the onset of low-grade inflammation in obese individuals [44]. Diets including high-fructose diets have a strong effect on the composition of the gut microbiota, resulting in increased gut permeability and systemic inflammation [45]. This low-grade systemic inflammation can activate the hepatic NF-κB through the IKK complex, which has been associated with the development of systemic and hepatic insulin resistance, MAFLD progression, and cardiovascular diseases [[43], [44], [45], [46]]. Our study suggests that the IKK complex is also engaged in the induction of simple steatosis and high plasma LDL cholesterol levels, a factor causally related to atherosclerotic cardiovascular disease (ASCVD) [47]. A part of our results is in line with a previous study showing that IKK-mediated NF-κB activation results in hepatic TG accumulation without liver inflammation and fibrosis [17]. Although both studies used an identical transgenic *Ikk2*ca mouse model, we found that liver TG and cholesterol were both elevated, whereas the hepatocyte-expressing IKKβca mice in the previous study by Lu et al. displayed only a modest accumulation of TG. Lu et al. explained this phenotype by an increased expression of lipogenic genes, including fatty acid synthase (*Fasn*) and stearol-CoA desaturase-1 (*Scd1*). However, we found no evidence for a significant change in the expression of genes associated with fatty acid or cholesterol metabolism. Instead, we showed that the IKK:NF-κB axis regulates these two pathways at a post-translational level. These phenotypic differences can likely be explained by the use of different diets, including the different chow diets because we observed Hep-IKKβca mice only developed steatosis on a carbohydrate-rich diet. Sugars, one of the main sources of carbohydrates in the diet, are associated with chronic inflammation and stimulate *DNL* and induce steatosis in mice and humans [48,49]. Supporting these findings, our WT control mice also developed liver steatosis after prolonged carbohydrate-rich diet feeding for ±40 weeks (Figure 7A). Furthermore, the importance of additional metabolic stresses to reveal metabolic differences is distinctly illustrated in HMGCR Ser871Ala mutant knock-in (KI) mice [50]. HMGCR is a downstream target of AMPK; phosphorylated active AMPK inhibits HMGCR activity by increasing its phosphorylation state at Ser871 [35,39]. HMGCR Ser871Ala mutant is insensitive for AMPK-mediated repression but the effect of this mutation on cholesterol synthesis was only seen when the mice were challenged with a carbohydrate-rich diet. Similar to our model, this metabolic stress led to elevated hepatic and plasma cholesterol levels, accompanied by increased hepatic TG levels caused by enhanced *de novo* lipogenesis.

The interaction between hepatic IKK activation and carbohydrates in the development of steatosis is substantiated by the observation that genetic ablation of the Toll-like receptor 4 (TLR4), a TLR that activates the IKK:NF-κB axis, protects mice from fructose-induced liver steatosis by an undefined mechanism [51,52]. Similarly, selective inhibition of tumor necrosis factor receptor 1 (TNFR1), another activator of the NF-κB signaling pathway, reduces liver steatosis in mice fed a high-fat diet supplemented with fructose and sucrose in the drinking water [53]. TNFR1 inhibition results in reduced expression of lipogenic genes due to decreased sterol regulatory element-binding protein 1 (SREBP1) expression and activation. We found that IKK-mediated NF-κB activation controls lipogenesis and cholesterol synthesis at a post-translation level, a type of regulation that has also been reported in a recent study [40] that reported that IKKβ augments lipid metabolism in liver tumors by promoting the stability of ACLY and FAS proteins, both key enzymes in lipogenesis. IKKβ stabilizes ACLY and FAS through phosphorylation of the deubiquitinase USP30, promoting deubiquitination of ACLY and FAS by USP30. We observed no changes in the protein levels of ACLY and FAS, indicating that under our conditions the IKK:NF-κB axis controls lipogenesis via an alternative manner. We showed that phosphorylation of AMPK, a key regulator of lipogenesis and cholesterol synthesis, was decreased in IKKβca;A20^LKO^ mice (Figure 6A). Phosphorylated AMPK inhibits DNL [37,54]. Increasing the phosphorylation level of AMPK by polyphenols, metformin, or expressing an active form of AMPK attenuates sugar-induced steatosis development [37,[53], [54], [55]], supporting the role of AMPK in carbohydrate-induced steatosis development. Although our data imply an essential role of AMPK activity, the downstream targets of AMPK, such as ACC1 and FAS, were not affected. This is an interesting observation as these proteins were also not affected in mouse livers expressing a constitutively active AMPK mutant (y1D136A) [37]. AMPK y1D136A mutant mice were protected against hepatic TG accumulation following a carbohydrate-rich diet, which was explained by decreased DNL due to increased phosphorylation of ACC1 [37]. Although *in vitro* data encouraged this model no *in vivo* evidence showed that ACC1 phosphorylation was correlated with decreased hepatic lipid accumulation [37]. In addition to ACC1, other proteins of the lipogenesis pathway may be post-translationally modified [40] and controlled by either AMPK or IKK activity under specific metabolic conditions (e.g., carbohydrate-rich diet).

As discussed above, HMGCR is a downstream target of AMPK and together with the elevated enzyme levels of HMGCS1, our data suggest that IKK-mediated NF-κB activation promotes cholesterol synthesis by increasing flux through the mevalonate pathway, also known as the HMG-CoA reductase pathway. This is an important finding because a recent study has shown an association of the activity of this pathway with the severity of MAFLD in humans [2]. Similar to our mouse model, decreased phosphorylation levels of AMPK and HMGCR were correlated with elevated cholesterol synthesis accompanied by high hepatic and plasma cholesterol. Presently, it remains unknown whether IKK directly regulates the mevalonate pathway or indirectly via AMPK. Several AMPK activators have been reported to investigate this but several effects induced by these activators could be explained by off-target effects or by AMPK activation in other organs/cells, which limit the use of AMPK activators to study the direct effect of AMPK activity on metabolic pathways [37,56]. The use of genetic mouse models, such as transgenic and tissue-specific knockout mice, can overcome this issue and are valuable to decipher the mechanism by which IKK regulates the mevalonate pathway. It is interesting to note that chronic hepatic activation of AMPK by expressing AMPK y1D136A mutant only decreases liver TG levels but not cholesterol levels following a carbohydrate-rich diet; it suggests that at certain metabolic conditions, HMGCR activity is not only controlled by AMPK [37], which may indicate that IKK directly controls the mevalonate pathway.

It remains poorly understood whether the metabolic effects are mediated by IKK or NF-κB activity, but because the NF-κB-mediated gene transactivation in our model is not further increased upon hepatocytic A20-deficiency and *A20* ablation alone does not result in steatosis, we speculate an essential role for the IKK complex in controlling DNL and cholesterol synthesis [40]. A20 acts upstream of IKK and despite the expression of a constitutively active IKKβ mutant, hepatocytic loss of A20 in Hep-IKKβca mice aggravates steatosis, which implies that A20 can mediate IKK functioning and its downstream effects via multiple mechanisms under specific conditions. A20 has shown modulating IKK activation via different ways using different proteins and post-translation modifications, such as linear ubiquitin chain assembly complex (LUBAC) and linear polyubiquitination [57]. In addition, other post-translational modifications of IKK components have also been reported [58]. Therefore, we speculate that stabilization and/or additional post-translational modifications of the IKK complex, which may be modulated by A20, control DNL and cholesterol synthesis in hepatocytes. Furthermore, in contrast to *IKBKB* (*IKKβ*), *A2*0 has been identified as a susceptibility locus for human immune and inflammatory diseases, such as rheumatoid arthritis, type 1 diabetes, coronary artery disease, and Crohn's disease [59]. However, associations of any of these gene polymorphisms with hepatic steatosis remain to be determined.

It has been shown that AMPK can inhibit NF-κB activity and, subsequently, inflammation in different immune cells [60]. Inflammation can also suppress AMPK activity in muscle, heart, adipose tissue, and macrophages by different unknown mechanisms [56,61]. For example, in muscle, inflammation induces protein phosphatase 2 (PP2C) expression and inactivates AMPK in a TNFR1-dependent manner [62]. TNF upregulates PP2C, likely through the IKK:NF-κB axis, we documented no difference in hepatic expression of PP2C between control and IKKβca;A20^LKO^ mice (Table S2). However, the role of other members of the serine/threonine protein phosphatases, such as *Ppmih* and *Ppmin*, which were both significantly upregulated (>2-fold) in IKKβca;A20^LKO^ mice (Table S2), in regulating AMPK activity remains unknown. Additional research is therefore required to provide better mechanistic insights by which the pro-inflammatory NF-κB signaling pathway in hepatocytes may control AMPK activity and the mevalonate pathway and, subsequently, MAFLD severity in patients [2].

Overall, we conclude that the hepatocytic IKK:NF-κB axis plays a critical role in the initiation and progression of MAFLD by controlling lipogenesis and cholesterol synthesis, thus it might contribute to cardiovascular disease risk in MAFLD patients. Although further research is needed, our data suggest that IKK-mediated NF-κB activation regulates the phosphorylation of AMPK and HMGCR and the protein expression of HMGCS1. A better understanding of the underlying mechanism could offer potential targets to prevent and treat MAFLD at several stages of its development, thereby mitigating cardiovascular risk.

## Appendix A Supplementary data

Multimedia component 1 Supplemental Figure 1. **Activation of the NF-κB signaling pathway in hepatocytes does not induce hepatic lipid accumulation or inflammation. (A)** Bodyweight, **(B)** liver weight, and **(C)** liver to body weight ratio of WT and Hep-IKKβca mice fed a standard chow diet (n = 6). **(D)** Hepatic triglyceride and cholesterol concentrations of WT and Hep-IKKβca mice. **(E)** H&E and ORO staining of livers of WT and Hep-IKKβca mice (n = 6). Representative images per group are shown. Scale bars represent 100 μm. Data are presented as mean ± SEM. Supplemental Figure 2. **Immunohistochemical characterization of livers from WT and Hep-IKKβca mice challenged with the****carbohydrate-rich diet. (A)** Representative immunohistochemical staining for the inflammatory markers F4/80, CD11b, B220, and CD3 of liver sections from WT and Hep-IKKβca mice fed the carbohydrate-rich diet, scale bars represent 100 μm **(B)** with quantification of the immunohistochemical staining (n = 6). **(C)** Heatmap presenting z-score normalized mRNA expression (determined by RNA-seq analysis) of hepatic gluconeogenic genes in WT and Hep-IKKβca mice fed the carbohydrate-rich diet (n = 6). Supplemental Figure 3. **Hepatic lipid accumulation in Hep-IKKβca mice is not caused by impaired β-oxidation (A)** Relative mRNA expression (determined by RNA-seq analysis) of genes related to beta-oxidation in WT and Hep-IKKβca mice fed the carbohydrate-rich diet (n = 6). **(B)** Gene set enrichment analysis results for the beta-oxidation category (raw data are shown in Supplemental Table 7). **(C)** Hepatic oxidative catabolism assessed by acetylcarnitine profiling using liquid chromatography with tandem mass spectrometry (n = 5–6). Data are presented as mean ± SEM, ∗P < 0.05, ∗∗P < 0.01 as determined by Student’s *t*-test. Supplemental Figure 4. **Immunohistochemical characterization of livers from WT and IKKβca;****A20**^**LKO**^**mice challenged with****the****carbohydrate-rich diet. (A)** Representative immunohistochemical staining for the inflammatory markers F4/80, CD11b, B220, and CD3 of liver sections from WT and IKKβca;A20^LKO^ mice fed the carbohydrate-rich diet, scale bars represent 100 μm **(B)** with quantification of the immunohistochemical staining (n = 6). **(C)** Hepatic triglyceride concentrations in WT and liver-specific *A20* knockout mice (A20^LKO^) fed the carbohydrate-rich diet (n = 5–7). **(D)** Relative mRNA expression of NF-κB gene and NF-κB target genes in livers of Hep-IKKβca and IKKβca;A20^LKO^ mice compared to their own WT controls as determined by RNA-seq analysis. Data are presented as mean ±SEM, ∗∗∗P < 0.001 as determined by Student’s *t*-test. Supplemental Figure 5. **Hepatocytic NF-κB activation in IKKβca;****A20**^**LKO**^**mice does not result in differential expression of lipogenic or cholesterogenic genes. (A)** Heatmap presenting z-score normalized mRNA expression (determined by RNA-seq analysis) of hepatic gluconeogenic genes, **(B)** fatty acid synthesis, and **(C)** cholesterol synthesis in WT and IKKβca;A20^LKO^ mice fed the carbohydrate-rich diet (n = 6). Supplemental Figure 6. **Hepatic protein levels of the lipoprotein receptors of WT and IKKβca;****A20**^**LKO**^**mice. (A)** Representative immunoblot and (**B**) quantification of the hepatic protein levels of LDLR, LRP1, and SR-B1 in WT and IKKβca;A20^LKO^ mice fed the carbohydrate-rich diet (n = 4). Data are presented as mean ± SEM, ∗P < 0.05 as determined by Student’s *t*-test. Supplemental Figure 7. ***De novo* lipogenesis and cholesterol synthesis in Hep-IKKβca mice and control mice. (A)** Absolute *de novo* synthesis of palmitate (C16:0), palmitoleic acid (C16:1), stearate (C18:0), and oleate (C18:1) from livers of WT and Hep-IKKβca mice (n = 10). **(B)** Relative hepatic cholesterol synthesis in WT and Hep-IKKβca mice (n=10). Data are presented as mean ± SEM, ∗∗P < 0.01 as determined by Student’s *t*-test. Supplemental Figure 8. **Hepatocytic activation of the NF-κB signaling pathway does not induce liver injury nor liver fibrosis in middle-aged mice. (A)** Bodyweight, **(B)** liver weight, **(C)** liver to body weight ratios, and **(D)** 6h fasted blood glucose levels of WT and IKKβca;A20^LKO^ mice (n = 7–12). **(E)** Relative hepatic mRNA levels of *Abcg5* and *Abcg8* in WT and IKKβca;A20^LKO^ mice as determined by qRT-PCR analysis. **(F)** Plasma bile acid concentrations in WT and IKKβca;A20^LKO^ mice. **(G)** Hepatic staining and **(H)** quantification of collagen fiber by Sirius-Red (SR) in livers of one-year-old WT and IKKβca;A20^LKO^ mice fed the carbohydrate-rich diet (n = 8–12). Representative images per group are shown. Scale bars represent 100 μm. **(I)** Plasma ALT and **(J)** AST levels in WT and IKKβca; A20^LKO^ mice. Data are presented as mean ± SEM. Multimedia component 1

## References

[1] Eslam M., Newsome P.N., Sarin S.K., Anstee Q.M., Targher G., Romero-Gomez M. (2020). A new definition for metabolic dysfunction-associated fatty liver disease: an international expert consensus statement. Journal of Hepatology.

[2] Min H., Kapoor A., Fuchs M., Mirshahi F., Zhou H., Maher J. (2012). Increased hepatic synthesis and dysregulation of cholesterol metabolism is associated with the severity of nonalcoholic fatty liver disease. Cell Metabolism.

[3] Ruissen M.M., Mak A.L., Beuers U., Tushuizen M.E., Holleboom A.G. (2020). Non-alcoholic fatty liver disease: a multidisciplinary approach towards a cardiometabolic liver disease. European Journal of Endocrinology.

[4] Haas J.T., Francque S., Staels B. (2016). Pathophysiology and mechanisms of nonalcoholic fatty liver disease. Annual Review of Physiology.

[5] Catrysse L., van Loo G. (2017). Inflammation and the metabolic syndrome: the tissue-specific functions of NF-κB. Trends in Cell Biology.

[6] He G., Karin M. (2011). NF-κB and STAT3- key players in liver inflammation and cancer. Cell Research.

[7] Sun B., Karin M. (2008). NF-κB signaling, liver disease and hepatoprotective agents. Oncogene.

[8] Ghosh S., Karin M. (2002). Missing pieces in the NF-κB puzzle. Cell.

[9] Bartuzi P., Hofker M.H., van de Sluis B. (2013). Tuning NF-κB activity: a touch of COMMD proteins. Biochimica et Biophysica Acta - Molecular Basis of Disease. Biochimica et Biophysica Acta.

[10] Israël A. (2010). The IKK complex, a central regulator of NF-kappaB activation. Cold Spring Harbor Perspectives in Biology.

[11] Riedlinger T., Liefke R., Meier-Soelch J., Jurida L., Nist A., Stiewe T. (2019). NF-κB p65 dimerization and DNA-binding is important for inflammatory gene expression. The FASEB Journal.

[12] Pasparakis M. (2009). Regulation of tissue homeostasis by NF-κB signalling: implications for inflammatory diseases. Nature Reviews Immunology.

[13] Farrell G.C., van Rooyen D., Gan L., Chitturi S. (2012). NASH is an inflammatory disorder: pathogenic, prognostic and therapeutic implications. Gut Liver.

[14] Popko K., Gorska E., Stelmaszczyk-Emmel A., Plywaczewski R., Stoklosa A., Gorecka D. (2010). Proinflammatory cytokines IL-6 and TNF-α and the development of inflammation in obese subjects. European Journal of Medical Research.

[15] Zeng T., Zhou J., He L., Zheng J., Chen L., Wu C. (2016). Blocking nuclear factor-kappa B protects against diet-induced hepatic steatosis and insulin resistance in mice. PLoS One.

[16] Bartuzi P., Wijshake T., Dekker D.C., Fedoseienko A., Kloosterhuis N.J., Youssef S.A. (2014). A cell-type-specific role for murine Commd1 in liver inflammation. Biochimica et Biophysica Acta - Molecular Basis of Disease.

[17] Lu H., Lei X., Zhang Q. (2015). Moderate activation of IKK2-NF-κB in unstressed adult mouse liver induces cytoprotective genes and lipogenesis without apparent signs of inflammation or fibrosis. BMC Gastroenterology.

[18] Ai L., Xu Q., Wu C., Wang X., Chen Z., Su D. (2015). A20 attenuates FFAs-induced lipid accumulation in nonalcoholic steatohepatitis. International Journal of Biological Sciences.

[19] Sasaki Y., Derudder E., Hobeika E., Pelanda R., Reth M., Rajewsky K. (2006). Canonical NF-κB activity, dispensable for B cell development, replaces BAFF-receptor signals and promotes B cell proliferation upon activation. Immunity.

[20] Vereecke L., Sze M., Mc Guire C., Rogiers B., Chu Y., Schmidt-Supprian M. (2010). Enterocyte-specific A20 deficiency sensitizes to tumor necrosis factor-induced toxicity and experimental colitis. Journal of Experimental Medicine.

[21] Kuipers F., van Ree J.M., Hofker M.H., Wolters W., In’t Veld G., Havinga R. (1996). Altered lipid metabolism in apolipoprotein E-deficient mice does not affect cholesterol balance across the liver. Hepatology.

[22] Laskewitz A.J., van Dijk T.H., Bloks V., Reijngoud D.J., van Lierop M.J., Dokter W.H. (2010). Chronic prednisolone treatment reduces hepatic insulin sensitivity while perturbing the fed-to-fasting transition in mice. Endocrinology.

[23] Kleiner D.E., Brunt E.M., van Natta M., Behling C., Contos M.J., Cummings O.W. (2005). Design and validation of a histological scoring system for nonalcoholic fatty liver disease. Hepatology.

[24] Bligh E.G., Dyer W.J. (1959). A rapid method of total lipid extraction and purification. Canadian Journal of Biochemistry and Physiology.

[25] Oosterveer M.H., van Dijk T.H., Tietge U.J.F., Boer T., Havinga R., Stellaard F. (2009). High fat feeding induces hepatic fatty acid elongation in mice. PLoS One.

[26] Renfurm L.N., Bandsma R.H.J., Verkade H.J., Hulzebos C.V., van Dijk T.H., Boer T. (2004). Cholesterol synthesis and de novo lipogenesis in premature infants determined by mass isotopomer distribution analysis. Pediatric Research.

[27] Derks T.G.J., Boer T.S., van Assen A., Bos T., Ruiter J., Waterham H.R. (2008). Neonatal screening for medium-chain acyl-CoA dehydrogenase (MCAD) deficiency in The Netherlands: the importance of enzyme analysis to ascertain true MCAD deficiency. Journal of Inherited Metabolic Disease.

[28] Wijers M., Zanoni P., Liv N., Vos D.Y., Jäckstein M.Y., Smit M. (2019). The hepatic WASH complex is required for efficient plasma LDL and HDL cholesterol clearance. JCI Insight.

[29] Mašek T., Filipović N., Vuica A., Starčević K. (2017). Effects of treatment with sucrose in drinking water on liver histology, lipogenesis and lipogenic gene expression in rats fed high-fiber diet. Prostaglandins Leukotrienes and Essential Fatty Acids.

[30] Cai D., Yuan M., Frantz D.F., Melendez P.A., Hansen L., Lee J. (2005). Local and systemic insulin resistance resulting from hepatic activation of IKK-beta and NF-kappaB. Nature Medicine.

[31] Beraza N., Malato Y., Vander Borght S., Liedtke C., Wasmuth H.E., Dreano M. (2008). Pharmacological IKK2 inhibition blocks liver steatosis and initiation of non-alcoholic steatohepatitis. Gut.

[32] Wartz I.E., O’Rourke K.M., Zhou H., Eby M., Aravind L., Seshagiri S. (2004). De-ubiquitination and ubiquitin ligase domains of A20 downregulate NF-κB signalling. Nature.

[33] Daemen S., Gainullina A., Kalugotla G., He L., Chan M.M., Beals J.W. (2021). Dynamic shifts in the composition of resident and recruited macrophages influence tissue remodeling in NASH. Cell Reports.

[34] van der Veen J.N., van Dijk T.H., Vrins C.L.J., van Meer H., Havinga R., Bijsterveld K. (2009). Activation of the liver X receptor stimulates trans-intestinal excretion of plasma cholesterol. Journal of Biological Chemistry.

[35] Clarke P.R., Hardie D.G. (1990). Regulation of HMG-CoA reductase: identification of the site phosphorylated by the AMP-activated protein kinase in vitro and in intact rat liver. The EMBO Journal.

[36] Lally J.S.V., Ghoshal S., DePeralta D.K., Moaven O., Wei L., Masia R. (2019). Inhibition of acetyl-CoA carboxylase by phosphorylation or the inhibitor ND-654 suppresses lipogenesis and hepatocellular carcinoma. Cell Metabolism.

[37] Woods A., Williams J.R., Muckett P.J., Mayer F.V., Liljevald M., Bohlooly M. (2017). Liver-specific activation of AMPK prevents steatosis on a high-fructose diet. Cell Reports.

[38] Esquejo R.M., Salatto C.T., Delmore J., Albuquerque B., Reyes A., Shi Y. (2018). Activation of liver AMPK with PF-06409577 corrects NAFLD and lowers cholesterol in rodent and primate preclinical models. EBioMedicine.

[39] Garcia D., Shaw R.J. (2017). AMPK: mechanisms of cellular energy sensing and restoration of metabolic balance. Molecular Cell.

[40] Gu L., Zhu Y., Lin X., Lu B., Zhou X., Zhou F. (2020). The IKKβ-USP30-ACLY Axis controls lipogenesis and tumorigenesis. Hepatology.

[41] Luedde T., Schwabe R.F. (2011). NF-κB in the liver-linking injury, fibrosis and hepatocellular carcinoma. Nature Reviews Gastroenterology & Hepatology.

[42] Baker R.G., Hayden M.S., Ghosh S. (2011). NF-κB, inflammation, and metabolic disease. Cell Metabolism.

[43] Cordeiro A., Costa R., Andrade N., Silva C., Canabrava N., Pena M.J. (2020). Does adipose tissue inflammation drive the development of non-alcoholic fatty liver disease in obesity?. Clinics and Research in Hepatology and Gastroenterology.

[44] Pereira S.S., Alvarez-Leite J.I. (2014). Low-grade inflammation, obesity, and diabetes. Current Obesity Reports.

[45] Cani P.D., Osto M., Geurts L., Everard A. (2012). Involvement of gut microbiota in the development of low-grade inflammation and type 2 diabetes associated with obesity. Gut Microbes.

[46] Wu H., Ballantyne C.M. (2020). Metabolic inflammation and insulin resistance in obesity. Circulation Research.

[47] Ference B.A., Ginsberg H.N., Graham I., Ray K.K., Packard C.J., Bruckert E. (2017). Low-density lipoproteins cause atherosclerotic cardiovascular disease. 1. Evidence from genetic, epidemiologic, and clinical studies. A consensus statement from the European Atherosclerosis Society Consensus Panel. European Heart Journal.

[48] Samuel V.T. (2011). Fructose induced lipogenesis: from sugar to fat to insulin resistance. Trends in Endocrinology and Metabolism.

[49] Lim J.S., Mietus-Snyder M., Valente A., Schwarz J.M., Lustig R.H. (2010). The role of fructose in the pathogenesis of NAFLD and the metabolic syndrome. Nature Reviews Gastroenterology and Hepatology.

[50] Loh K., Tam S., Murray-Segal L., Huynh K., Meikle P.J., Scott J.W. (2019). Inhibition of adenosine monophosphate–activated protein kinase–3-hydroxy-3-methylglutaryl coenzyme A reductase signaling leads to hypercholesterolemia and promotes hepatic steatosis and insulin resistance. Hepatology Communications.

[51] Kawai T., Akira S. (2007). Signaling to NF-κB by toll-like receptors. Trends in Molecular Medicine.

[52] Spruss A., Kanuri G., Wagnerberger S., Haub S., Bischoff S.C., Bergheim I. (2009). Toll-like receptor 4 is involved in the development of fructose-induced hepatic steatosis in mice. Hepatology.

[53] Wandrer F., Liebig S., Marhenke S., Vogel A., John K., Manns M.P. (2020). TNF-Receptor-1 inhibition reduces liver steatosis, hepatocellular injury and fibrosis in NAFLD mice. Cell Death & Disease.

[54] Li Y., Xu S., Mihaylova M.M., Zheng B., Hou X., Jiang B. (2011). AMPK phosphorylates and inhibits SREBP activity to attenuate hepatic steatosis and atherosclerosis in diet-induced insulin-resistant mice. Cell Metabolism.

[55] Karise I., Ornellas F., Barbosa-da-Silva S., Matsuura C., Del Sol M., Aguila M.B. (2017). Liver and Metformin: lessons of a fructose diet in mice. Biochimie Open.

[56] Day E.A., Ford R.J., Steinberg G.R. (2017). AMPK as a therapeutic target for treating metabolic diseases. Trends in Endocrinology and Metabolism.

[57] Verhelst K., Carpentier I., Kreike M., Meloni L., Verstrepen L., Kensche T. (2012). A20 inhibits LUBAC-mediated NF-κB activation by binding linear polyubiquitin chains via its zinc finger 7. The EMBO Journal.

[58] Perkins N.D. (2006). Post-translational modifications regulating the activity and function of the nuclear factor kappa B pathway. Oncogene.

[59] Catrysse L., Vereecke L., Beyaert R., Loo G. van. (2014). A20 in inflammation and autoimmunity. Trends in Immunology.

[60] O'Neill L.A.J., Grahame Hardie D. (2013). Metabolism of inflammation limited by AMPK and pseudo-starvation. Nature.

[61] Jeon S.M. (2016). Regulation and function of AMPK in physiology and diseases. Experimental & Molecular Medicine.

[62] Steinberg G.R., Michell B.J., van Denderen B.J.W., Watt M.J., Carey A.L., Fam B.C. (2006). Tumor necrosis factor alpha-induced skeletal muscle insulin resistance involves suppression of AMP-kinase signaling. Cell Metabolism.

