# Supplementary material for: The hepatocyte IKK:NF-κB axis promotes liver steatosis by stimulating de novo lipogenesis and cholesterol synthesis
Source: Mol Metab. 2021 Oct 6;54:101349. doi: 10.1016/j.molmet.2021.101349 (PMC8581577; doi:10.1016/j.molmet.2021.101349)
Supplement: Multimedia component 1 — Supplemental Figure 1. Activation of the NF-κB signaling pathway in hepatocytes does not induce hepatic lipid accumulation or inflammation. (A) Bodyweight, (B) liver weight, and (C) liver to body weight ratio of WT and Hep-IKKβca mice fed a standard chow diet (n = 6). (D) Hepatic triglyceride and cholesterol concentrations of WT and Hep-IKKβca mice. (E) H&E and ORO staining of livers of WT and Hep-IKKβca mice (n = 6). Representative images per group are shown. Scale bars represent 100 μm. Data are presented as mean ± SEM. Supplemental Figure 2. Immunohistochemical characterization of livers from WT and Hep-IKKβca mice challenged with thecarbohydrate-rich diet. (A) Representative immunohistochemical staining for the inflammatory markers F4/80, CD11b, B220, and CD3 of liver sections from WT and Hep-IKKβca mice fed the carbohydrate-rich diet, scale bars represent 100 μm (B) with quantification of the immunohistochemical staining (n = 6). (C) Heatmap presenting z-score normalized mRNA expression (determined by RNA-seq analysis) of hepatic gluconeogenic genes in WT and Hep-IKKβca mice fed the carbohydrate-rich diet (n = 6). Supplemental Figure 3. Hepatic lipid accumulation in Hep-IKKβca mice is not caused by impaired β-oxidation (A) Relative mRNA expression (determined by RNA-seq analysis) of genes related to beta-oxidation in WT and Hep-IKKβca mice fed the carbohydrate-rich diet (n = 6). (B) Gene set enrichment analysis results for the beta-oxidation category (raw data are shown in Supplemental Table 7). (C) Hepatic oxidative catabolism assessed by acetylcarnitine profiling using liquid chromatography with tandem mass spectrometry (n = 5–6). Data are presented as mean ± SEM, ∗P < 0.05, ∗∗P < 0.01 as determined by Student’s t-test. Supplemental Figure 4. Immunohistochemical characterization of livers from WT and IKKβca;A20LKOmice challenged withthecarbohydrate-rich diet. (A) Representative immunohistochemical staining for the inflammatory markers F4/80, CD11b, B220, and [file mmc1.zip › Supplemental Table 3.docx]

**Supplemental Table 3.** List of antibodies

| **Antibody** | **Company** | **Catalogue#** | **Dilution** |
| --- | --- | --- | --- |
| ACC | Cell Signaling | 3676 | 1:1000 |
| ACC^ser79^ | Cell Signaling | 3661 | 1:1000 |
| ACLY | Cell Signaling | 13390 | 1:1000 |
| α-Tubulin | Cell Signaling | 2144 | 1:1000 |
| AMPK | Cell Signaling | 2532 | 1:1000 |
| AMPK^Thr172*^ | Cell Signaling | 2531 | 1:1000 |
| β-actin | Sigma | A5441 | 1:1000 |
| FAS | Cell Signaling | 3180 | 1:1000 |
| FLAG M2-peroxidase | Sigma | A8592 | 1:1000 |
| HMGCR | Thermo scientific | PA537367 | 1:1000 |
| HMGCR^Ser872*^ | Bioss | bs-4063R | 1:1000 |
| HMGCS1 | Cell Signalling | 42201 | 1:1000 |
| HSP90 | Cell Signalling | 4874 | 1:1000 |
| Lamin A/C | Cell Signalling | 2032 | 1:1000 |
| LDLR | Abnova | PAB8804 | 1:1000 |
| LRP1 | Abcam | ab92544 | 1:1000 |
| p65 | Cell Signalling | 4764 | 1:1000 |
| SR-B1 | Novus biologicals | NB400-131 | 1:1000 |
| Goat anti-mouse IgG (H+L)-HRP conjugate | Bio-Rad | 1706516 | 1:10000 |
| Goat anti-rabbit IgG (H+L)-HRP conjugate | Bio-Rad | 1706515 | 1:10000 |
| B220 | Santa Cruz | Sc-19597 | 1:100 |
| Cd3 | Dako | A0452 | 1:250 |
| Cd11b | Abcam | Ab133357 | 1:6000 |
| F4/80 | Abcam | Ab6640 | 1:500 |
| Goat anti-rabbit/biotin | Vector | BA-1000 | 1:250 |
| Goat anti-rat/biotin | Vector | BA-9400 | 1:125 |

* To detect AMPK^Thr172^ and HMGCR^Ser872*^ a 1:2000 dilution of the 2^nd^ antibody has been used.
