# Supplementary material for: The hepatocyte IKK:NF-κB axis promotes liver steatosis by stimulating de novo lipogenesis and cholesterol synthesis
Source: Mol Metab. 2021 Oct 6;54:101349. doi: 10.1016/j.molmet.2021.101349 (PMC8581577; doi:10.1016/j.molmet.2021.101349)
Supplement: Multimedia component 1 — Supplemental Figure 1. Activation of the NF-κB signaling pathway in hepatocytes does not induce hepatic lipid accumulation or inflammation. (A) Bodyweight, (B) liver weight, and (C) liver to body weight ratio of WT and Hep-IKKβca mice fed a standard chow diet (n = 6). (D) Hepatic triglyceride and cholesterol concentrations of WT and Hep-IKKβca mice. (E) H&E and ORO staining of livers of WT and Hep-IKKβca mice (n = 6). Representative images per group are shown. Scale bars represent 100 μm. Data are presented as mean ± SEM. Supplemental Figure 2. Immunohistochemical characterization of livers from WT and Hep-IKKβca mice challenged with thecarbohydrate-rich diet. (A) Representative immunohistochemical staining for the inflammatory markers F4/80, CD11b, B220, and CD3 of liver sections from WT and Hep-IKKβca mice fed the carbohydrate-rich diet, scale bars represent 100 μm (B) with quantification of the immunohistochemical staining (n = 6). (C) Heatmap presenting z-score normalized mRNA expression (determined by RNA-seq analysis) of hepatic gluconeogenic genes in WT and Hep-IKKβca mice fed the carbohydrate-rich diet (n = 6). Supplemental Figure 3. Hepatic lipid accumulation in Hep-IKKβca mice is not caused by impaired β-oxidation (A) Relative mRNA expression (determined by RNA-seq analysis) of genes related to beta-oxidation in WT and Hep-IKKβca mice fed the carbohydrate-rich diet (n = 6). (B) Gene set enrichment analysis results for the beta-oxidation category (raw data are shown in Supplemental Table 7). (C) Hepatic oxidative catabolism assessed by acetylcarnitine profiling using liquid chromatography with tandem mass spectrometry (n = 5–6). Data are presented as mean ± SEM, ∗P < 0.05, ∗∗P < 0.01 as determined by Student’s t-test. Supplemental Figure 4. Immunohistochemical characterization of livers from WT and IKKβca;A20LKOmice challenged withthecarbohydrate-rich diet. (A) Representative immunohistochemical staining for the inflammatory markers F4/80, CD11b, B220, and [file mmc1.zip › Supplemental Table 7.docx]

**Supplemental Table 7**. Gene set enrichment analysis results for data beta-oxidation category in livers of Hep-IKKβca mice.

|  | **SYMBOL** | **RANK IN GENE LIST** | **RANK METRIC SCORE** | **RUNNING ES** | **CORE ENRICHMENT** |
| --- | --- | --- | --- | --- | --- |
| 1 | [CNR1](https://ensembl.org/Search/Results?q=CNR1) | 149 | 3.075 | 0.1019 | No |
| 2 | [TWIST1](https://ensembl.org/Search/Results?q=TWIST1) | 442 | 2.119 | 0.1681 | No |
| 3 | [ABCD2](https://ensembl.org/Search/Results?q=ABCD2) | 2500 | 1.101 | 0.1614 | No |
| 4 | [ECI3](https://ensembl.org/Search/Results?q=ECI3) | 3714 | 0.874 | 0.1652 | No |
| 5 | [ACACB](https://ensembl.org/Search/Results?q=ACACB) | 5018 | 0.729 | 0.1621 | No |
| 6 | [ACAT2](https://ensembl.org/Search/Results?q=ACAT2) | 5335 | 0.685 | 0.1787 | No |
| 7 | [ACOX2](https://ensembl.org/Search/Results?q=ACOX2) | 7464 | 0.461 | 0.1486 | No |
| 8 | [ACSL5](https://ensembl.org/Search/Results?q=ACSL5) | 8855 | 0.367 | 0.1312 | No |
| 9 | [ECHS1](https://ensembl.org/Search/Results?q=ECHS1) | 9772 | 0.304 | 0.1219 | No |
| 10 | [ABCD1](https://ensembl.org/Search/Results?q=ABCD1) | 10384 | 0.264 | 0.1178 | No |
| 11 | [DBI](https://ensembl.org/Search/Results?q=DBI) | 10390 | 0.264 | 0.1267 | No |
| 12 | [EHHADH](https://ensembl.org/Search/Results?q=EHHADH) | 10467 | 0.261 | 0.1340 | No |
| 13 | [CRAT](https://ensembl.org/Search/Results?q=CRAT) | 12078 | 0.222 | 0.1069 | No |
| 14 | [ABCD4](https://ensembl.org/Search/Results?q=ABCD4) | 12495 | 0.196 | 0.1046 | No |
| 15 | [HADH](https://ensembl.org/Search/Results?q=HADH) | 14204 | 0.116 | 0.0718 | No |
| 16 | [PEX2](https://ensembl.org/Search/Results?q=PEX2) | 14567 | 0.097 | 0.0673 | No |
| 17 | [BDH2](https://ensembl.org/Search/Results?q=BDH2) | 15077 | 0.069 | 0.0587 | No |
| 18 | [SESN2](https://ensembl.org/Search/Results?q=SESN2) | 15255 | 0.059 | 0.0570 | No |
| 19 | [ACAA1A](https://ensembl.org/Search/Results?q=ACAA1A) | 15462 | 0.047 | 0.0541 | No |
| 20 | [ECHDC1](https://ensembl.org/Search/Results?q=ECHDC1) | 15789 | 0.028 | 0.0481 | No |
| 21 | [ACAT3](https://ensembl.org/Search/Results?q=ACAT3) | 16102 | 0.009 | 0.0417 | No |
| 22 | [ACOXL](https://ensembl.org/Search/Results?q=ACOXL) | 16398 | 0.000 | 0.0353 | No |
| 23 | [ACSBG2](https://ensembl.org/Search/Results?q=ACSBG2) | 17161 | 0.000 | 0.0189 | No |
| 24 | [ETFA](https://ensembl.org/Search/Results?q=ETFA) | 38401 | -0.005 | -0.4383 | No |
| 25 | [ACADS](https://ensembl.org/Search/Results?q=ACADS) | 38418 | -0.006 | -0.4384 | No |
| 26 | [ETFDH](https://ensembl.org/Search/Results?q=ETFDH) | 38527 | -0.014 | -0.4402 | No |
| 27 | [PEX7](https://ensembl.org/Search/Results?q=PEX7) | 38541 | -0.016 | -0.4400 | No |
| 28 | [ACADL](https://ensembl.org/Search/Results?q=ACADL) | 38825 | -0.034 | -0.4449 | No |
| 29 | [ACOX3](https://ensembl.org/Search/Results?q=ACOX3) | 38829 | -0.034 | -0.4438 | No |
| 30 | [AKT1](https://ensembl.org/Search/Results?q=AKT1) | 38891 | -0.038 | -0.4438 | No |
| 31 | [ACADM](https://ensembl.org/Search/Results?q=ACADM) | 39238 | -0.058 | -0.4493 | No |
| 32 | [MTOR](https://ensembl.org/Search/Results?q=MTOR) | 39512 | -0.075 | -0.4526 | Yes |
| 33 | [AUH](https://ensembl.org/Search/Results?q=AUH) | 39537 | -0.076 | -0.4505 | Yes |
| 34 | [HADHB](https://ensembl.org/Search/Results?q=HADHB) | 39560 | -0.078 | -0.4484 | Yes |
| 35 | [PEX5](https://ensembl.org/Search/Results?q=PEX5) | 39608 | -0.081 | -0.4466 | Yes |
| 36 | [MLYCD](https://ensembl.org/Search/Results?q=MLYCD) | 39851 | -0.098 | -0.4485 | Yes |
| 37 | [LONP2](https://ensembl.org/Search/Results?q=LONP2) | 39944 | -0.104 | -0.4469 | Yes |
| 38 | [PLIN5](https://ensembl.org/Search/Results?q=PLIN5) | 39999 | -0.107 | -0.4444 | Yes |
| 39 | [ACAT1](https://ensembl.org/Search/Results?q=ACAT1) | 40002 | -0.107 | -0.4408 | Yes |
| 40 | [IVD](https://ensembl.org/Search/Results?q=IVD) | 40078 | -0.113 | -0.4385 | Yes |
| 41 | [CROT](https://ensembl.org/Search/Results?q=CROT) | 40192 | -0.120 | -0.4368 | Yes |
| 42 | [ECHDC2](https://ensembl.org/Search/Results?q=ECHDC2) | 40381 | -0.134 | -0.4363 | Yes |
| 43 | [AKT2](https://ensembl.org/Search/Results?q=AKT2) | 40418 | -0.137 | -0.4324 | Yes |
| 44 | [CPT2](https://ensembl.org/Search/Results?q=CPT2) | 40678 | -0.153 | -0.4327 | Yes |
| 45 | [HADHA](https://ensembl.org/Search/Results?q=HADHA) | 40713 | -0.156 | -0.4281 | Yes |
| 46 | [TYSND1](https://ensembl.org/Search/Results?q=TYSND1) | 41050 | -0.180 | -0.4292 | Yes |
| 47 | [HSD17B10](https://ensembl.org/Search/Results?q=HSD17B10) | 41265 | -0.193 | -0.4272 | Yes |
| 48 | [ECI1](https://ensembl.org/Search/Results?q=ECI1) | 41320 | -0.199 | -0.4215 | Yes |
| 49 | [MFSD2A](https://ensembl.org/Search/Results?q=MFSD2A) | 42320 | -0.237 | -0.4350 | Yes |
| 50 | [SLC25A17](https://ensembl.org/Search/Results?q=SLC25A17) | 42347 | -0.240 | -0.4273 | Yes |
| 51 | [DECR1](https://ensembl.org/Search/Results?q=DECR1) | 42654 | -0.273 | -0.4246 | Yes |
| 52 | [CPT1A](https://ensembl.org/Search/Results?q=CPT1A) | 42690 | -0.280 | -0.4157 | Yes |
| 53 | [ACAA2](https://ensembl.org/Search/Results?q=ACAA2) | 42758 | -0.288 | -0.4074 | Yes |
| 54 | [HSD17B4](https://ensembl.org/Search/Results?q=HSD17B4) | 42876 | -0.300 | -0.3996 | Yes |
| 55 | [GCDH](https://ensembl.org/Search/Results?q=GCDH) | 43172 | -0.337 | -0.3944 | Yes |
| 56 | [SCP2](https://ensembl.org/Search/Results?q=SCP2) | 43184 | -0.339 | -0.3831 | Yes |
| 57 | [ACAD11](https://ensembl.org/Search/Results?q=ACAD11) | 43266 | -0.350 | -0.3729 | Yes |
| 58 | [ETFB](https://ensembl.org/Search/Results?q=ETFB) | 43487 | -0.379 | -0.3647 | Yes |
| 59 | [IRS2](https://ensembl.org/Search/Results?q=IRS2) | 43640 | -0.409 | -0.3539 | Yes |
| 60 | [ABCD3](https://ensembl.org/Search/Results?q=ABCD3) | 43928 | -0.433 | -0.3453 | Yes |
| 61 | [ACADVL](https://ensembl.org/Search/Results?q=ACADVL) | 43958 | -0.440 | -0.3309 | Yes |
| 62 | [ETFBKMT](https://ensembl.org/Search/Results?q=ETFBKMT) | 44042 | -0.455 | -0.3171 | Yes |
| 63 | [IRS1](https://ensembl.org/Search/Results?q=IRS1) | 44398 | -0.522 | -0.3069 | Yes |
| 64 | [SLC27A2](https://ensembl.org/Search/Results?q=SLC27A2) | 44818 | -0.637 | -0.2942 | Yes |
| 65 | [ACAA1B](https://ensembl.org/Search/Results?q=ACAA1B) | 44837 | -0.638 | -0.2727 | Yes |
| 66 | [ACOX1](https://ensembl.org/Search/Results?q=ACOX1) | 45103 | -0.736 | -0.2533 | Yes |
| 67 | [ECI2](https://ensembl.org/Search/Results?q=ECI2) | 45316 | -0.768 | -0.2316 | Yes |
| 68 | [FABP1](https://ensembl.org/Search/Results?q=FABP1) | 46215 | -1.397 | -0.2032 | Yes |
| 69 | [LEP](https://ensembl.org/Search/Results?q=LEP) | 46277 | -1.502 | -0.1531 | Yes |
| 70 | [ADIPOQ](https://ensembl.org/Search/Results?q=ADIPOQ) | 46500 | -4.622 | 0.0001 | Yes |
