# Supplementary material for: The hepatocyte IKK:NF-κB axis promotes liver steatosis by stimulating de novo lipogenesis and cholesterol synthesis
Source: Mol Metab. 2021 Oct 6;54:101349. doi: 10.1016/j.molmet.2021.101349 (PMC8581577; doi:10.1016/j.molmet.2021.101349)
Supplement: Multimedia component 1 — Supplemental Figure 1. Activation of the NF-κB signaling pathway in hepatocytes does not induce hepatic lipid accumulation or inflammation. (A) Bodyweight, (B) liver weight, and (C) liver to body weight ratio of WT and Hep-IKKβca mice fed a standard chow diet (n = 6). (D) Hepatic triglyceride and cholesterol concentrations of WT and Hep-IKKβca mice. (E) H&E and ORO staining of livers of WT and Hep-IKKβca mice (n = 6). Representative images per group are shown. Scale bars represent 100 μm. Data are presented as mean ± SEM. Supplemental Figure 2. Immunohistochemical characterization of livers from WT and Hep-IKKβca mice challenged with thecarbohydrate-rich diet. (A) Representative immunohistochemical staining for the inflammatory markers F4/80, CD11b, B220, and CD3 of liver sections from WT and Hep-IKKβca mice fed the carbohydrate-rich diet, scale bars represent 100 μm (B) with quantification of the immunohistochemical staining (n = 6). (C) Heatmap presenting z-score normalized mRNA expression (determined by RNA-seq analysis) of hepatic gluconeogenic genes in WT and Hep-IKKβca mice fed the carbohydrate-rich diet (n = 6). Supplemental Figure 3. Hepatic lipid accumulation in Hep-IKKβca mice is not caused by impaired β-oxidation (A) Relative mRNA expression (determined by RNA-seq analysis) of genes related to beta-oxidation in WT and Hep-IKKβca mice fed the carbohydrate-rich diet (n = 6). (B) Gene set enrichment analysis results for the beta-oxidation category (raw data are shown in Supplemental Table 7). (C) Hepatic oxidative catabolism assessed by acetylcarnitine profiling using liquid chromatography with tandem mass spectrometry (n = 5–6). Data are presented as mean ± SEM, ∗P < 0.05, ∗∗P < 0.01 as determined by Student’s t-test. Supplemental Figure 4. Immunohistochemical characterization of livers from WT and IKKβca;A20LKOmice challenged withthecarbohydrate-rich diet. (A) Representative immunohistochemical staining for the inflammatory markers F4/80, CD11b, B220, and [file mmc1.zip › Supplemental Table 4.docx]

**Supplemental Table 4**. Histological scoring for NASH activities in the livers of WT and Hep-IKKβca mice.

| **genotype** | | **age** | **steatosis** | **lobular Inflammation** | **portal Inflammation** | **oval cell proliferation** | **NAS** |
| --- | --- | --- | --- | --- | --- | --- | --- |
| Hep-IKKβca | WT | 20 weeks | 1.00 | 1.80 | 0.40 | 1.20 | 3.20 |
|  | TG | 20 weeks | 2.33 | 2.50 | 0.83 | 1.83 | 5.17 |
